# Brain atrophy staging in spinocerebellar ataxia type 3 for clinical prognosis and trial enrichment

**DOI:** 10.1016/j.ebiom.2025.106090

**Published:** 2025-12-23

**Authors:** Hannah Baumeister, Philipp Wegner, Mónica Ferreira, Tamara Schaprian, Marcondes C. França, Thiago Junqueira Ribeiro Rezende, Alberto Rolim Muro Martinez, Hong Jiang, Zhao Chen, Liao Weihua, Marcus Grobe-Einsler, Berkan Koyak, Demet Önder, Bart van de Warrenburg, Judith van Gaalen, Alexandra Durr, Giulia Coarelli, Matthis Synofzik, Ludger Schöls, Paola Giunti, Hector Garcia-Moreno, Gülin Öz, James Joers, Dagmar Timmann, Andreas G. Thieme, Heike Jacobi, Jeroen de Vries, Peter Barker, Chiadikaobi Onyike, Eva-Maria Ratai, Jeremy D. Schmahmann, Kathrin Reetz, Jon Infante, Jeannette Huebener-Schmid, David Kuegler, Thomas Klockgether, David Berron, Jennifer Faber, DELCODE/DANCERai, Falk Lüsebrink, Stefan Hetzer, Michael Ewers, Julian Hellmann-Regen, Eike Spruth, Daniel Janowitz, Ingo Kilimann, Marie T. Kronmüller, Annika Spottke, Oliver Peters, Josef Priller, Katharina Buerger, Stefan Teipel, Frank Jessen, Emrah Düzel, Anna Gamez, Hannah Asperger, Okka Kimmich, Gabor C. Petzold, ESMI MRI Study Groupsai, Kennet Teichmann, Sarah Bernsen, Katharina Hill, Ilse Willemse, Teije van Prooije, Friedrich Erdlenbruch, Thomas Ernst, Benjamin Bender, Johann E. Jende, Khalaf Bushara, Leire Manrique, Pauline Lallemant-Dudek, Sandro Romanzetti, Alexander Lange, Maya Shrestha, Anton Ludwig, Alena Rosenow, Tim Elter, Magda M. Santana, Eberhard Pracht, Tony Stoecker

**Affiliations:** a German Center for Neurodegenerative Diseases (DZNE), Magdeburg, Germany; b German Center for Neurodegenerative Diseases (DZNE), Bonn, Germany; c University of Bonn, Bonn, Germany; d Department of Neurology, School of Medical Sciences, University of Campinas (UNICAMP), Campinas, Brazil; e Department of Neurology, Xiangya Hospital, Central South University, Changsha, Hunan, China; f Key Laboratory of Hunan Province in Neurodegenerative Disorders, Central South University, Changsha, Hunan, China; g National Clinical Research Center for Geriatric Disorders, Xiangya Hospital, Central South University, Changsha, Hunan, China; h Radiological Intervention Center, Department of Radiology, Xiangya Hospital, Central South University, Changsha, Hunan, China; i Center for Neurology, Department of Parkinson, Sleep and Movement Disorders, University Hospital Bonn, Bonn, Germany; j Department of Neurology, Donders Institute for Brain, Cognition, and Behaviour, Radboud University Medical Center, Nijmegen, the Netherlands; k Department of Neurology, Rijnstate Hospital, Arnhem, Nijmegen, the Netherlands; l Sorbonne Université, Paris Brain Institute - ICM, Inserm, CNRS, APHP, Pitié-Salpêtrière University Hospital, Paris, France; m Division Translational Genomics of Neurodegenerative Diseases, Hertie Institute for Clinical Brain Research and Center of Neurology, University of Tübingen, Tübingen, Germany; n German Center for Neurodegenerative Diseases (DZNE), Tübingen, Germany; o Department of Neurology and Hertie-Institute for Clinical Brain Research, University of Tübingen, Tübingen, Germany; p Ataxia Centre, Department of Clinical and Movement Neurosciences, UCL Queen Square Institute of Neurology, London, UK; q Center for Magnetic Resonance Research, Department of Radiology, University of Minnesota, Minneapolis, MN, USA; r Department of Neurology and Center for Translational Neuro- and Behavioral Sciences, University of Duisburg-Essen, Essen, Germany; s Department of Neurology, University Hospital Heidelberg, Heidelberg, Germany; t Department of Neurology, University Medical Center Groningen, University of Groningen, Groningen, the Netherlands; u Johns Hopkins University School of Medicine, Baltimore, MD, USA; v Department of Psychiatry and Behavioral Sciences, Johns Hopkins University School of Medicine, Baltimore, MD, USA; w Department of Radiology, Massachusetts General Hospital, Boston, MA, USA; x A. A. Martinos Center for Biomedical Imaging and Harvard Medical School Charlestown, Charlestown, MA, USA; y Ataxia Center, Laboratory for Neuroanatomy and Cerebellar Neurobiology, Department of Neurology, Massachusetts General Hospital and Harvard Medical School, Boston, MA, USA; z Department of Neurology, RWTH Aachen University, Aachen, Germany; aa JARA-Brain Institute Molecular Neuroscience and Neuroimaging, Forschungszentrum Jülich, Jülich, Germany; ab Neurology Service, University Hospital Marqués de Valdecilla-IDIVAL, Universidad de Cantabria, Centro de Investigación en Red de Enfermedades Neurodegenerativas (CIBERNED), Santander, Spain; ac Institute of Medical Genetics and Applied Genomics, University of Tübingen, Tübingen, Germany; ad Centre for Rare Diseases, University of Tübingen, Tübingen, Germany; ae Clinical Memory Research Unit, Department of Clinical Sciences Malmö, Lund University, Lund, Sweden; af Center for Behavioral Brain Sciences (CBBS), Otto-von-Guericke University, Magdeburg, Germany; ag Department of Neuroradiology, University Hospital Bonn, Bonn, Germany

**Keywords:** Disease progression modelling, Movement disorders, Imaging biomarker, Machine learning, Ataxia

## Abstract

**Background:**

Spinocerebellar ataxia type 3 (SCA3) is characterised by progressive brain atrophy, with regional volume loss detectable via MRI prior to clinical manifestation. We aimed to identify the previously unknown sequence of brain atrophy in SCA3 and evaluate whether this sequence can be translated into an atrophy staging framework to enable accurate clinical prognosis and trial enrichment.

**Methods:**

We included data from 322 SCA3 mutation carriers, enrolled in observational studies conducted across Europe, the Americas, and Asia. Participants underwent follow-up assessments up to five years after baseline. The Subtype and Stage Inference machine learning algorithm was applied to estimate the most likely atrophy sequence(s) from baseline anatomical MRI. The Scale for the Assessment and Rating of Ataxia (SARA) was used to capture ataxia severity. Atrophy stages were analysed in relation to SARA and time from disease onset. Interventional trials were simulated to estimate required sample sizes under different atrophy stage eligibility criteria.

**Findings:**

We identified a uniform sequence of brain atrophy in SCA3, characterised by earliest volumetric decline in the caudal brainstem and substantial involvement of the white matter. Atrophy stage was associated with both SARA and time from disease onset. Atrophy staging outperformed single-region volumetrics in predicting SARA over time. Applying atrophy stage cut-offs substantially reduced the sample sizes needed to adequately power hypothetical clinical trials.

**Interpretation:**

These findings yield mechanistic insights into the progression of neurodegeneration in SCA3 and possess immediate translational relevance, facilitating patient stratification and sample enrichment for interventional trials.

**Funding:**

10.13039/100002243National Ataxia Foundation (NAF).

**Research in context**

**Evidence before this study**

We searched Medline and ISI Web of Science for reports published before April 25, 2025, with the search terms [“spinocerebellar ataxia type 3” OR “SCA3” AND “atrophy” OR “MRI” AND “prospective” OR “follow-up” OR “longitudinal”] plus [“spinocerebellar ataxia type 3” OR “SCA3” AND “SuStaIn” OR “Subtype and Stage Inference” OR “event-based modelling” OR “event-based modelling”]. Only peer-reviewed, English-language reports of human cohort studies with at least ten participants were considered. Seven independent studies with participant numbers ranging from 17 to 23 and follow-up times from six months to 5 years as well as one study with an overlapping sample found progressive atrophy of a number of brain structures and cervical spinal cord, as well as increasing abnormalities of diffusion parameters of a number of brain fibre tracts in SCA3 mutation carriers compared to healthy controls.

**Added value of this study**

In this global MRI study, we investigated the progression of regional brain volume loss in a large cohort of SCA3 mutation carriers both before and after gait ataxia onset. Using data-driven disease progression modelling, we reconstructed the sequence of brain atrophy in SCA3, arising from the brainstem and involving extensive white matter atrophy. Our data enable the definition of distinct brain atrophy stages that correlate with disease severity and duration, predict future clinical decline, and support refined patient stratification.

**Implications of all the available evidence**

The identified, purely data-driven sequence of brain atrophy captures the specific temporal order and dynamics of regional volume loss in SCA3. The derived brain atrophy stages improve the prediction of clinical progression beyond established clinical assessments and other MRI-based markers. In clinical trials, atrophy staging enables a substantial reduction in required sample size with only a minimal increase in screen failure rates, thus providing an effective enrichment strategy for future targeted trials.

## Introduction

Spinocerebellar ataxia type 3 (SCA3) is the most common autosomal dominantly inherited ataxia worldwide with a symptom onset in adult life. Clinical hallmarks are a progressive loss of balance and coordination accompanied by slurred speech, leading to disability and premature death.^1^^,^^2^ SCA3 is a neurodegenerative condition caused by unstable expansions of polyglutamine encoding CAG repeats, resulting in the formation of an abnormally elongated disease protein.^3^ Targeted therapies are being developed, and first safety trials of antisense oligonucleotides (ASOs) have been initiated (https://clinicaltrials.gov, NCT05160558, NCT05822908). Assuming safety and tolerability, such therapies offer a promising opportunity for preventive treatment aimed at postponing the onset of clinical symptoms and, consequently, delaying full clinical manifestation. Meanwhile, these advances create an urgent need for biomarkers that facilitate patient identification and disease monitoring in the crucial period imminent to symptom manifestation. Cranial MRI is routinely used in SCA3 to assess atrophy as a downstream biomarker of neurodegeneration. Atrophy typically affects the brainstem, cerebellum, and basal ganglia, where earliest volumetric abnormalities are detectable before clinical symptoms emerge.^4^, ^5^, ^6^, ^7^ However, the exact order and temporal dynamics of earliest brain atrophy due to SCA3 remain unknown. In fact, atrophy may also progress heterogeneously, unfolding along multiple, spatiotemporally distinct cascades.

Data-driven models of disease progression are capable of resolving the sequence of pathological changes in neurodegenerative diseases like SCA3.^8^ This is done through the integration of various biomarker information, such as different volumetric MRI readouts, into a high-level framework of disease progression.^8^ When applied to MRI data, these approaches not only offer mechanistic insights into neurodegeneration progression but also enable the staging of individual patients within a modelled atrophy sequence. Such atrophy staging has the potential of enhancing the use of MRI in various contexts. In clinical practice, it may enhance the assessment of current disease severity or improve prognostic accuracy. In pharmacological trials, atrophy staging may enable prognostic sample enrichment by increasing the proportion of individuals with greater risk of worsening during the trial period, leading to more efficient trials with greater statistical power.^9^^,^^10^

Several statistical approaches, including the Subtype and Stage Inference (SuStaIn) machine learning algorithm, have been designed to capture progression patterns in neurodegenerative diseases characterised by gradual pathological changes, as seen in SCA3.^11^ Unlike similar approaches, SuStaIn integrates classical event-based modelling—an established method for analysing progressive change—with statistical clustering. This allows SuStaIn to infer the most likely sequence of pathological events from cross-sectional data and to detect distinct progression subtypes, capturing potential disease heterogeneity.^11^^,^^12^

In the present study, we aimed to establish a staging framework that effectively captures earliest atrophy in SCA3 using SuStaIn modelling of volumetric MRI data. To explore its potential clinical relevance, we examined the clinical correlates of individual atrophy stages and their use for predicting future ataxia severity. To ensure applicability beyond the training data, we performed rigorous cross-validation to evaluate the robustness of the inferred atrophy sequences, individual stage assignments, and predictions of ataxia trajectories over time. Finally, we simulated interventional trials to estimate the potential of atrophy stage as a prognostic enrichment variable.

## Methods

### Participants

We included 322 SCA3 mutation carriers and 100 healthy controls from observational studies conducted at 21 academic hospitals in Europe, the Americas, and Asia (Supplementary Table S1). Participants were enrolled between September 16, 2004, and June 19, 2020 in the retrospective studies and between January 26, 2017, and June 20, 2023 in the prospective studies. For all SCA3 participants, the diagnosis was genetically confirmed through either diagnostic genetic testing or centralised genetic testing within the study setting. Information on concomitant diseases, pharmacological treatments, and rehabilitation therapies was not systematically available. Healthy controls were recruited from the community (with no history of neurological or psychiatric diseases), were unrelated family members (e.g., spouses), or, in the case of relatives at risk for SCA3, were only included after confirmation of a negative genetic test result for SCA3.

Participants were included in the present study if anatomical MRI data were available and passed visual quality control. Healthy controls were included for age- and sex-adjusted scaling of regional brain volumes and were not further analysed.

### Ethics

All participants provided written informed consent in accordance with the Declaration of Helsinki. Ethical approval was obtained from the institutional review boards of all participating sites. In the United Kingdom (London), approval was granted by the London–Chelsea Research Ethics Committee (REC reference: 17/LO/0381; approval date: April 28, 2017). In Germany (Bonn), approval was issued by the Ethics Committee of the Medical Faculty, University of Bonn (reference: 176/16; approval date: October 11, 2016; Amendment 1: September 17, 2020; Amendment 2: August 27, 2024). In the Netherlands (Nijmegen), approval was obtained from the CMO Regio Arnhem-Nijmegen for two studies: Cerebellar dysfunction and its compensation in presymptomatic carriers of dominant ataxia genes (local reference: 2010/438; national reference: NL34874.091.10; approval date: January 10, 2011) and European Spinocerebellar Ataxia Type 3/Machado-Joseph Disease (ESMI) (local reference: 2016-2554; national reference: NL25267.091.16; approval date: April 3, 2017). In Spain (Santander), approval was granted by the Comité de Ética de la Investigación con medicamentos de Cantabria (reference: 2018.282; approval dates: February 1, 2019, and April 26, 2021 for Amendment 1). In the United States (Minnesota), approval was obtained from the Institutional Review Board of the University of Minnesota (IRB study number: 0502M67488; approval date: June 9, 2017). In France (Paris), approval was issued by the Comité de Protection des Personnes SUD-EST IV (protocol number: RBM 01-29; NCT00140829; IDRCB No. 2021-A00989-32; first patient enrolled: February 10, 2004; study completion: 2020). In Brazil (Campinas), approval was obtained from the Institutional Review Board of the University of Campinas (CAAE: 83241318.3.1001.5404). In China (Changsha), approval was obtained from the Ethics Committee of Xiangya Hospital (Central South University, reference: 202310206).

### Procedures

The Scale for the Assessment and Rating of Ataxia (SARA) was used to assess ataxia presence (threshold at SARA ≥3, otherwise we use the term “preataxic”) and severity.^13^^,^^14^ SARA was available longitudinally in 35 preataxic and 95 ataxic individuals, from a mean of 2.06 (*SD* = 1.26) follow-up assessments (mean follow-up interval = 1.20 [*SD* = 0.51] years). Neurological signs other than ataxia were assessed using the Inventory of Non-Ataxia Signs (INAS).^15^ Clinical disease stages were determined through self-reported gait status, distinguishing individuals as follows: no gait difficulties (“normal”), gait disturbances (“gait disturbance”), requiring a walking aid or support (“walking aid”), and permanent wheelchair use (“wheelchair”).^16^ CAG repeat lengths of the expanded and normal alleles were determined at the Institute for Medical Genetics and Applied Genomics of the University of Tübingen, Germany (*n* = 98, 30.43%), or taken from medical records. Time from disease onset was calculated from the reported first occurrence of gait disturbances and estimated for individuals without this information (Supplementary Methods).

Cranial T1-weighted MRI scans underwent visual quality control. Most included scans (*n* = 316, 74.9% across healthy controls and SCA3 mutation carriers) were acquired with a 1 mm isotropic resolution. The remaining scans were resampled to a 1 mm isotropic resolution. Images were N4 bias field corrected (Advanced Normalisation Tools, v2.1).^17^ Regional brain volumes were determined using FastSurfer and CerebNet.^5^^,^^18^ FreeSurfer v6.0 was used to obtain regional volumes within the brainstem.^19^ All hemispheric volumes were summed. Follow-up MRI was available for 19 preataxic and 53 ataxic SCA3 mutation carriers, with an average of 1.97 (*SD* = 1.23) follow-up scans (mean follow-up interval = 1.20 [*SD* = 0.50] years).

SuStaIn was implemented to identify potential atrophy sequence(s) from baseline volumetric data using python v3.9. Details on model specification and selection are given in the Supplementary Methods. To maximise sensitivity to early disease progression, we restricted the model to regions previously reported to show volumetric decline already in preataxic SCA3, being medulla oblongata, pons, midbrain, pallidum, superior cerebellar peduncle, cerebellar white matter, as well as the flocculonodular and anterior lobe of the cerebellum.^4^, ^5^, ^6^^,^^20^ With healthy controls as reference, these volumes were corrected for their relationship with intracranial volume and converted to age- and sex-adjusted *w*-scores, reflecting the adjusted *SD* differences from healthy controls.^21^ We set *w*∈{−1,−2,−3} as atrophy thresholds of increasing volume loss below −1, −2 and −3 *SD*, respectively, and estimated *k*∈{1,2,3,4,5} possible sequences of 24 atrophy events (three *w*-thresholds × eight regional volumes). We refer to the positions of these events within the event sequence as atrophy stages. Though continuous modelling occurred across atrophy stages, we note that their scaling does not imply equidistant time intervals. A ten-fold cross-validation scheme guided model selection and evaluation (Supplementary Methods). The selected atrophy progression model, entirely trained on baseline data, was applied to follow-up data to investigate stage progression and adherence to the model's inherent assumption of stage monotonicity (i.e., non-declining stages over time).

### Statistics

Statistical analyses were conducted in R v4.2.2. The threshold for statistical significance was set at *p* < .05. False discovery rate (FDR) correction was applied where required.

Robustness of the best-fit atrophy sequence across cross-validation folds was quantified using mean Bhattacharyya coefficients ($\bar{BC}$). For reference, this metric was also calculated based on randomised sequences (${\bar{BC}}_{0}$; Supplementary Methods). Model generalisability was evaluated by comparing atrophy stages from model training based on all available data to their cross-validated out-of-sample predictions using absolute errors. Change in atrophy stage over time was estimated via linear mixed effects models (LMMs) with participant-level random slopes and intercepts. Potential effects of scanner manufacturer and field strength were assessed using multiple regression models, with atrophy stage as the outcome, scanner manufacturer and field strength as predictors of interest, and time from disease onset included as a covariate to account for the tendency of specific sites to contribute cases at different disease stages.

To examine their cross-sectional clinical correlates, ANCOVA with Tukey *post-hoc* tests compared atrophy stages by ataxia and gait status, correcting for age and sex. Multiple regression models including the same covariates examined whether time from onset predicted atrophy stage and whether atrophy stage predicted SARA scores and INAS counts. These relationships were assessed by ataxia status through interaction terms, except when predicting SARA scores, where separate models were used per group to avoid endogenous stratification.

To estimate the prognostic properties of atrophy stages in comparison to other volumetric MRI readouts, we used LMMs with participant-wise random slopes and intercepts to predict longitudinal SARA scores. We defined a covariates-only model with baseline age, sex, CAG repeat length, INAS count, and gait status and their interactions with continuous time as fixed effects (“clinical reference model”). We specified further models by adding different *w*-scored MRI readouts and their interactions with time as predictors to the clinical reference model. Specifically, we defined models to include (i) atrophy stage, (ii) single individual volumes (e.g., pons volume), (iii) all individual volumes as separate predictors (“all volumes”), or (iv) the sum of all individual volumes (“global ROI volume”) as MRI readouts. The MRI readout × time interaction effect was the primary predictor of interest across models, as it tested the prognostic properties of the respective MRI measure for SARA trajectories. All predictors were modelled linearly. To provide standardised regression coefficients (*β*), numeric predictors were *z*-scored relative to baseline observations. Model comparisons were performed using the Akaike Information Criterion (AIC). Differences in AIC (ΔAIC) were interpreted according to established guidelines, with ΔAIC ≤ −2 indicating little support for the higher-AIC model, and ΔAIC ≤ −10 indicating essentially no empirical support for the higher-AIC model.^22^ Furthermore, we used ten-repeat ten-fold cross-validation to estimate each model's prediction accuracy, expressed as non-standardised root mean square errors (RMSEs).^23^ To evaluate the contribution of baseline MRI readouts, we extracted each participant's final available follow-up prediction within each out-of-sample fold. We then compared RMSEs of all models including MRI readouts with those of the clinical reference model using Student's *t*-tests, applying a correction proposed by Nadeau and Bengio to account for the interdependent sample structure inherent to cross-validation designs.^24^

Finally, we simulated two-year, placebo-controlled, parallel-group clinical trials (80% power), collecting SARA scores quarterly as the primary endpoint. We determined the required sample size and proportion of additional screen failures (percent of participants excluded from baseline sample) for trials with lower atrophy stage limits from 0 to *j*, where *j* is the maximum cut-off that retains at least 15 individuals in the current sample. Calculations were based on LMMs evaluating treatment effects on SARA progression (effect sizes 20–50% slowing) using the “longpower” R package.^25^

### Role of funders

The funder of the study had no role in study design, data collection, data analysis, data interpretation, or writing of the report.

## Results

Baseline characteristics of the SCA3 sample as well as data availability are summarised in Table 1 and Supplementary Figure S1. Healthy controls had a mean age of 43.92 (*SD* = 13.86) years, with 50% females (as indicated by self-report). Details on the statistical correction of regional volumes for intracranial volume as well as the *w*-scoring procedure are provided in Supplementary Tables S2 and S3.

**Table 1:** Characteristics of the analysed sample of SCA3 mutation carriers.

Variable	Missing (%)	Preataxic SCA3 (*n* = 67)	Ataxic SCA3 (*n* = 255)	Ataxic *versus* preataxic SCA3
Age (years)		37.54 (9.14)	47.69 (11.55)	*t* (127.21) = 7.63∗∗∗∗
Sex^a^				*Χ*^2^(1) = 2.96 (n.s.)
Female		39 (58.21%)	116 (45.49%)	
Male		28 (41.76%)	138 (54.51%)	
CAG expanded allele	46 (14.29%)	68.00 (65.00–70.00)	70.00 (67.00–72.00)	*t* (117.45) = 6.15∗∗∗∗
Time from disease onset (years)	3 (0.93%)	−7.55 (−11.80 to 1.90)	9.00 (5.57–12.86)	*t* (78.73) = 14.26∗∗∗∗
SARA score	6 (1.86%)	1.50 (0.50–2.50)	12.00 (9.00–15.50)	*t* (296.37) = 28.87∗∗∗∗
INAS count	114 (35.40%)	1.00 (1.00–2.00)	4.00 (3.00–6.00)	*t* (126.27) = 12.07∗∗∗∗
Self-reported gait status	175 (54.35%)			*Χ*^2^(3) = 83.77∗∗∗∗
Normal		22 (64.71%)	1 (0.88%)	
Gait difficulties		12 (35.29%)	72 (63.72%)	
Walking aid		0 (0.00%)	34 (30.09%)	
Wheelchair		0 (0.00%)	6 (5.31%)	
At least one follow-up visit		19 (28.36%)	53 (20.78%)	

Data are *n* (%), mean (*SD*), or median (IQR).

∗∗∗∗*p* < .0001.

Abbreviation: n.s., non-significant.

a Sex was self-reported.

### Regional brain atrophy in SCA3 progresses along a single uniform sequence

**Fig. 1:**
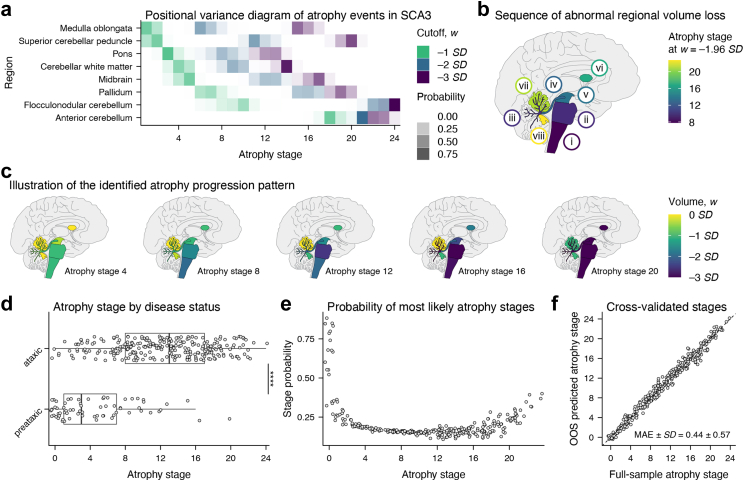
**The proposed model of atrophy progression in SCA3, derived from structural MRI data from 322 SCA3 mutation carriers.** (**a**) The sequence of atrophy events as well as the model's certainty about event locations is illustrated through a positional variance diagram. Higher opaqueness represents a higher certainty of an atrophy event occurring in the respective location within the sequence. The term “atrophy event” here refers to the point at which a regional volume falls below a specified threshold, with different colours representing different thresholds. (**b**) The sequence at which regional volumes are abnormally reduced, as indicated by a value of 1.96 *SD* below the mean of the healthy control group after correcting for the effects of age and sex. The pictograms shown in (**c**) depict the cumulative regional volume loss across the modelled atrophy stages. (**d**) Ataxic patients were assigned significantly higher atrophy stages than preataxic mutation carriers. (**e**) Probabilities of patients' most likely atrophy stages were lowest at intermediate stages and increased towards the endpoints of the sequence. (**f**) Atrophy stages from full-sample model training and their cross-validated out-of-sample (OOS) predictions showed high agreement. All atrophy stage data points are jittered by ± 0.5 along the respective axis to improve readability. ∗∗∗∗*p* < .0001.

We identified a single spatiotemporal atrophy sequence that best fit our dataset of SCA3 mutation carriers without evidence of any alternative cascades (Fig. 1a–c; Supplementary Figure S2). Fig. 1b shows the order in which regional volumes reach abnormality in this sequence, defined by a *w*-score of −1.96 (i.e., outside the central 95% range of the healthy population), revealing an atrophy pattern ascending from the brainstem with early involvement of white matter. The pons and cerebellar white matter exhibited the most rapid progression, being the first regions where volumes dropped below −3 *SD*. We found no significant effects of scanner manufacturer or field strength on atrophy stage, although non-significant trends suggested slightly higher stages in data acquired on 1.5 T and GE scanners (Supplementary Table S4).

Atrophy stages were significantly higher in ataxic than in preataxic SCA3 mutation carriers (*F* (1, 318) = 85.10, *p* < .0001; Fig. 1d). Staging certainty was highest at the sequence's endpoints (Fig. 1e). The identified atrophy sequence remained robust across cross-validation folds, suggesting its generalisability to other samples (mean ± *SD BC*_CV_ = 0.91 ± 0.01; mean ± *SD BC*_random_ = 0.54 ± 0.03; Supplementary Figure S3). Comparing full-sample model stages with their cross-validated out-of-sample predictions indicated precise and robust stage assignments outside the training data (MAE ± *SD* = 0.44 ± 0.57; Fig. 1f; Supplementary Figure S3). Bland-Altman analysis revealed that this robustness was independent of atrophy severity (Supplementary Figure S4).

**Fig. 2:**
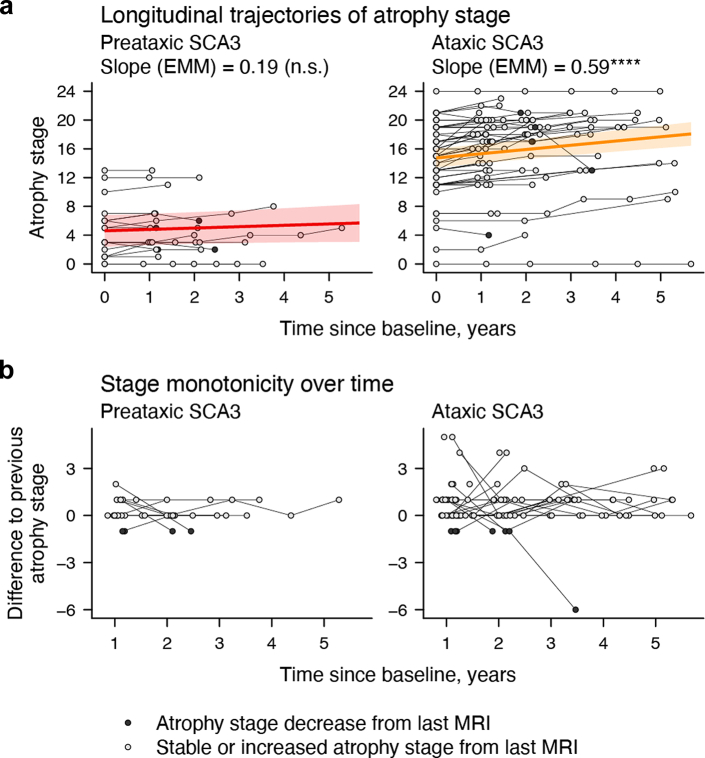
**Development of atrophy stages over time.** (**a**) On the group level, atrophy stages significantly increased over time in ataxic (*n* = 158), but not preataxic (*n* = 56), SCA3 mutation carriers. The displayed parameters are the estimated marginal means (EMMs) of the linear trends of atrophy stage over time for preataxic and ataxic SCA3 mutation carriers. (**b**) The differences in atrophy stage to the previous MRI show that violations of stage monotonicity (decreasing stages over time) were rare and primarily occurred as a decrease of one stage. ∗∗∗∗*p* < .0001.

The trained model was next used to determine atrophy stages from follow-up MRI scans. Atrophy stage significantly increased by an estimated rate of 0.49 stages per year (95% confidence interval [C.I.] = [0.32; 0.66], *p* < .001). Modelling these trends by ataxia status, we found significant longitudinal increases in ataxic (estimated marginal mean of the linear trend [EMM_trend_] = 0.59, 95% C.I. = [0.39; 0.79], *p* < .0001) but not in preataxic mutation carriers (EMM_trend_ = 0.19, 95% C.I. = [–0.15; 0.53], *p* = .2666; Fig. 2a; Supplementary Tables S5 and S6). Yet, there was a substantial portion of scans assigned a higher atrophy stage than the respective previous scan in both groups (32.43% [*n* = 12] in preataxic, 54.29% [*n* = 57] in ataxic SCA3). A decrease in atrophy stage—violating the assumption of stage monotonicity—occurred in 10.81% (*n* = 4) and 6.67% (*n* = 7) of follow-up scans of preataxic and ataxic patients, respectively. Most of these cases (*n* = 10, 90.91%) reflected a minor decrease of one atrophy stage (Fig. 2b).

### Brain atrophy stage correlates with disease duration and ataxia severity

**Fig. 3:**
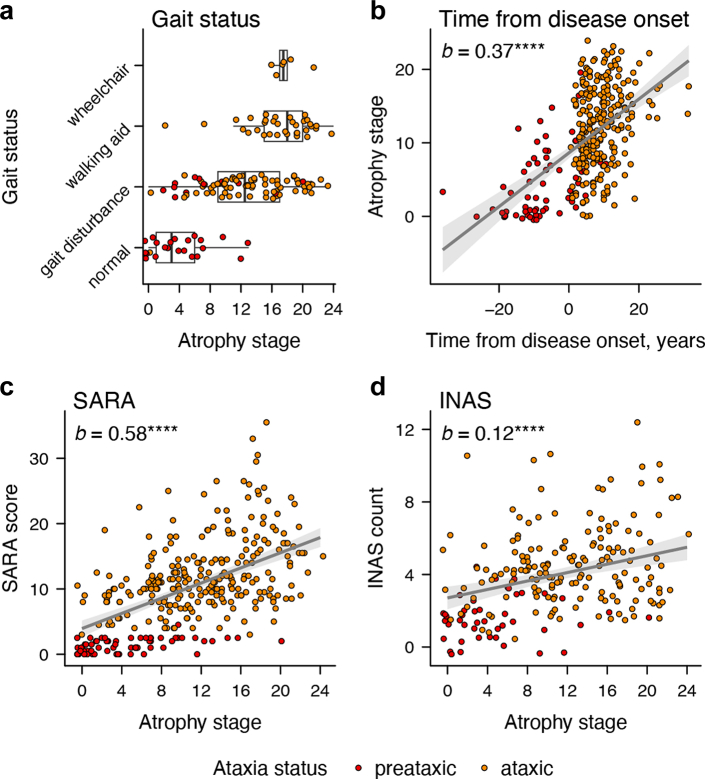
**Cross-sectional clinical correlates of atrophy stage.** Plots illustrate (**a**) atrophy stages across self-reported gait impairment (*n* = 147), as well as the associations of atrophy stage with (**b**) estimated time from disease onset (*n* = 319), (**c**) SARA scores (*n* = 316), and (**d**) INAS counts (*n* = 208) for the two SCA3 groups. The regression lines in (**b–d**) display the estimated relationships, averaging across sexes and setting age to the median of all included SCA3 mutation carriers (=46 years). Ribbons indicate 95% C.I.s. The displayed parameter *b* is the unstandardised estimate. All atrophy stage and INAS count data points are jittered by ± 0.5 along the respective axis to improve readability. ∗∗∗∗*p* < .0001.

We next assessed the cross-sectional clinical correlates of atrophy stage. We identified a significant relationship of gait status on atrophy stage, where atrophy stage increased with worsening gait (*F* (1, 141) = 26.43, *p* < .0001). Only walking aid and wheelchair users did not show a significant difference (b = 0.78, *p* .7211, range of all other *b* = 4.39–13.34, *p*_FDR_ ≤ 0.0171; Fig. 3a; Supplementary Tables S7 and S8). Time from disease onset predicted atrophy stage in the whole sample (*b* = 0.37, *β* = 0.55, *p* < .0001; Fig. 3b). This effect did not differ by ataxia status (*b* = 0.05, *β* = 0.08, *p* = .5586) and was significant both in preataxic (EMM_trend_ = 0.21, standardised EMM_trend_ = 0.32, *p* = .0037) and ataxic individuals (EMM_trend_ = 0.27, standardised EMM_trend_ = 0.40, *p* < .0001). Atrophy stage was associated with higher SARA scores in the whole sample (*b* = 0.58, *β* = 0.53, *p* < .0001; Fig. 3c), with this relationship being significant in both the preataxic (*b* = 0.09, *β* = 0.43, *p* = .0003) and ataxic (*b* = 0.40, *β* = 0.39, *p* < .0001) subgroups. The relationship with INAS count was positive in the whole sample (*b* = 0.12, *β* = 0.32, *p* < .0001; Fig. 3d) but did not differ by ataxia status (*b* = 0.01, *β* = 0.03, *p* = .8718) and was non-significant when estimated in either of the groups separately (*p*_preataxic_ = 0.5206; *p*_ataxic_ = 0.0531). All model characteristics are reported in Supplementary Tables S9–S12.

### Brain atrophy staging outperforms volumetric MRI measures for clinical prognosis

**Fig. 4:**
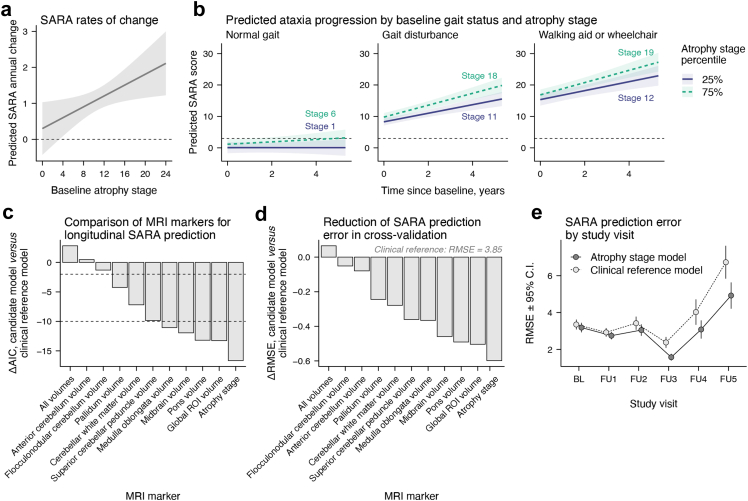
**Predicting ataxia trajectories with baseline atrophy stage using linear mixed-effects modelling.** (**a**) Higher baseline atrophy stage was associated with a faster increase in SARA scores in 63 SCA3 mutation carriers. This relationship was adjusted for baseline age, sex, gait status, INAS count, and length of the CAG expanded allele. (**b**) Using the same model, we predicted longitudinal SARA scores as a function of baseline gait status and atrophy stage percentile. The dashed line indicates a SARA score of 3, representing the threshold for manifest ataxia. Ribbons indicate 95% C.I.s. For the shown predictions, continuous covariates were set to the median of the respective gait status level and effects were averaged across sexes. (**c**) ΔAIC between models including different MRI markers and the covariates-only clinical reference model indicate that adding atrophy stage as a predictor results in the greatest improvement in model efficiency. The dashed lines represent established thresholds for interpreting ΔAIC. ΔAIC ≤ −2 indicates little support for the clinical reference model and ΔAIC ≤ −10 indicates essentially no empirical support for the clinical reference model. (**d**) The cross-validated Root Mean Squared Error (RMSE) was used to evaluate the performance of LMMs in predicting SARA scores at each participant's maximum follow-up assessment, based on different baseline MRI markers. Displayed are ΔRMSEs relative to the clinical reference model. (**e**) RMSEs of the model including atrophy stage *versus* the clinical reference model including no MRI information are shown by study visit. For visualisation purposes, RMSEs are displayed by study visit (mean follow-up interval = 1.20 [*SD* = 0.50]), though time was modelled continuously in the underlying LMMs. Abbreviations: BL, baseline. FU, follow-up.

Next, we investigated if atrophy stages could improve the prognosis of SARA trajectories over time. Adding atrophy stage to the clinical reference model substantially improved model fit (ΔAIC = −16.65), indicating added prognostic value beyond baseline demographics and clinical characteristics. Crucially, higher baseline atrophy stages were linked to faster ataxia progression (*β*_time × stage_ = 0.08, *p* = .0168). Using this model, we predicted annual SARA change by baseline atrophy stage (Fig. 4a) and SARA trajectories by baseline gait status and atrophy stage (Fig. 4b). Atrophy stage outperformed other MRI markers (Fig. 4c) and was superior to the second best model in terms of AIC (ΔAIC = −3.39). Moreover, atrophy stage showed the strongest time × MRI marker interaction effect (Supplementary Table S13). Cross-validation revealed that adding atrophy stage to the clinical reference model reduced error in predicting longitudinal SARA scores at each participant's maximum visit (Fig. 4d, RMSE_stage_ = 3.25, RMSE_reference_ = 3.85). This 15.58% reduction was the largest among all candidate models and was statistically significant before (*t* = 2.08, *p*_raw_ = 0.0398) but not after FDR-correction (*p*_FDR_ = 0.2063). No other model reached statistical significance at the uncorrected or corrected level (Supplementary Table S14). Examined by study visit, the benefit of including baseline atrophy stage as a predictor was observable at any time point but numerically increased with longer follow-up duration (Fig. 4e, see Supplementary Figure S5 for other MRI markers).

### Brain atrophy staging enables prognostic enrichment in clinical trials

**Fig. 5:**
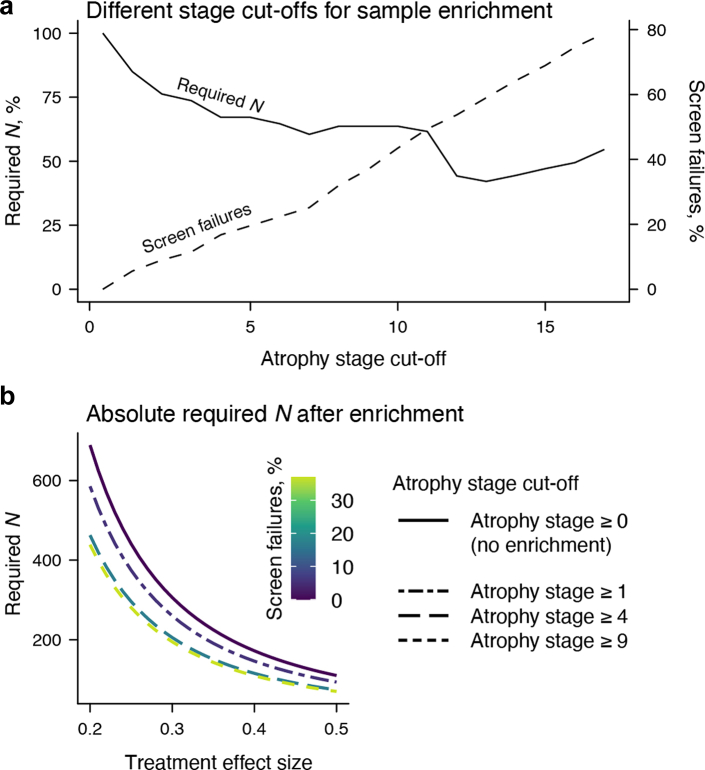
**Sample size reductions for hypothetical two-year, placebo-controlled, parallel-group trial using atrophy stage as an enrichment variable.** (**a**) Expected reductions in sample size (*N*; left *y*-axis) and corresponding changes in screen failures rates (right *y*-axis) for cut-offs between atrophy stages 0 and 17. Higher cut-offs were not modelled due to limited data availability. (**b**) Absolute required *N* for four enrichment scenarios: no enrichment (including all participants regardless of atrophy stage) and progressively excluding individuals below atrophy stages 1, 4, and 9. *N* represents the total number of participants across both study arms, assuming equal allocation per arm.

Our final analysis assessed the value of using atrophy stage as a criterion for enriching clinical trial samples. Fig. 5a illustrates how different atrophy stage cut-offs affect required sample size and screen failure rates, with lower cut-offs offering an especially favourable cost-benefit ratio. For example, including only individuals at atrophy stage ≥4 yielded a screen failure rate of 16.77% while reducing the sample size by 32.86%. The required sample sizes for this and other illustrative cut-offs are detailed in Fig. 5b. Notably, adopting a more conservative cut-off (e.g., atrophy stage 9) had little effect on the required sample size but inflated screen failures. Excluding individuals without relevant atrophy (stage 0) already reduced sample size by 15.03% with minimal additional screen failures (5.59%). This enrichment effect remained robust when trial eligibility was further restricted by age (25–60 years) and SARA score (3–18), as done in a currently ongoing trial (NCT05822908; Supplementary Figure S6).

## Discussion

Using machine learning-based disease progression modelling, the present study uncovered the cascade of early brain atrophy in a uniquely large dataset of SCA3 mutation carriers representing a broad range of the disease course. Individual atrophy stages showed significant cross-sectional associations with both time from disease onset and ataxia severity. Outperforming other MRI markers, atrophy stages were robust predictors of ataxia trajectories over time. Finally, we demonstrated that this prognostic value can be utilised to implement sample enrichment in interventional trials, leading to a considerable reduction in needed sample sizes.

Our data-driven results indicate that SCA3 follows one uniform pattern of atrophy evolution, setting it apart from the heterogeneity seen in other neurodegenerative diseases like Alzheimer's disease or frontotemporal dementia.^11^^,^^12^ This may be explained by the single genetic cause of SCA3 but is not self-evident, as various clinical subtypes of SCA3 have been described.^26^, ^27^, ^28^, ^29^ Moreover, heterogeneity in other neurodegenerative diseases may be facilitated by age-related co-pathologies, which are less common in the mostly middle-aged patients with SCA3.

The atrophy progression sequence identified in SCA3 reveals a pattern ascending from the lower brainstem via the pons and cerebellar white matter to basal ganglia, with late involvement of the cerebellar cortex. This sequence, with a start in the medulla oblongata, suggests that the origin of pathology of SCA3 possibly lies in the spinal cord. The available data did not permit a reliable assessment of the spinal cord. Thus, further MRI studies incorporating the spinal cord in SCA3 are needed. The most rapid progression up to three age- and sex-adjusted *SD* below healthy controls was observed in the pons and cerebellar white matter, which is in line with a previous study in a partially overlapping sample, where volumes of these regions showed the highest sensitivity to change in SCA3.^20^

Notably, the identified sequence follows anatomical connections. In principle, this observation is compatible with transneuronal spreading of an abnormal disease protein, as put forward in other neurodegenerative diseases.^30^ To date, there is no clear evidence of a spreading pathology in SCAs.^31^ Meanwhile, these anatomical connections are formed by white matter tracts and there is increasing evidence for a crucial role of oligodendroglia in SCA3.^32^ Beyond impaired maturation, molecular signatures in SCA3 mouse models have been linked not only to neuroaxonal but also to oligodendroglial pathology,^33^^,^^34^ highlighted further by reversed MR spectroscopy profiles following ASO treatment.^35^ Although a direct link to oligodendroglia-specific pathology remains to be demonstrated, human SCA3 studies have revealed widespread white matter involvement, using similar MR spectroscopy profiles as well as diffusion tensor imaging, complemented by recent evidence of cerebellar lipid dysregulation in SCA3.^4^^,^^20^^,^^36^^,^^37^

Our study suggests that the proposed atrophy progression framework can be generalised to independent data, as indicated by a reliable recovery of the atrophy sequence across cross-validation folds. This conclusion is supported by the high agreement between stages derived from full-sample training and cross-validation. While staging confidence tended to be lower for mid-range stages, we observed high agreement irrespective of atrophy severity, indicating that reliable staging is possible across the disease course.^38^

Overall clinical deterioration, reflected by clinical milestones of gait deterioration, was associated with higher atrophy stages, except in the latest stage (wheelchair users). This group included only six participants, likely biasing the sample toward less severely affected individuals still able to attend study visits. In both preataxic and ataxic participants, atrophy stage was associated with time from ataxia onset and SARA scores, indicating that atrophy stage reflects both temporal and clinical disease progression. The robustness of this result in only preataxic individuals highlights the potential use of atrophy staging for capturing sub-clinical disease progression. Consequently, our model may aid the stratification of preataxic mutation carriers with imminent symptom onset; a promising window for effective disease-modifying intervention. In contrast, we found weaker associations with non-ataxia signs. This is unsurprising, given that the used INAS count combines a heterogenous range of signs, including those that are due to peripheral neuropathy.

Investigating the prognostic value of atrophy stage, we found that a higher baseline stage was associated with faster increases in SARA scores, outperforming all other evaluated MRI measures. While sample size limitations prevented a separate analysis of pre-ataxic individuals, our longitudinal predictions suggest a significant annual increase in SARA scores from atrophy stage four onward. This stage or higher was observed in 47.76% of pre-ataxic mutation carriers at baseline, placing them at an increased risk of SARA progression. Cross-validation indicated that including atrophy stage reduced out-of-sample prediction error by 15.58% compared with a reference model based on non-MRI clinical and demographic information, representing the numerically largest reduction among the evaluated MRI readouts. While this effect did not survive correction for multiple comparisons, the substantial effect size and significant uncorrected *p*-value in this exploratory analysis suggest, with appropriate caution, that integrating multiple volumetric markers within a unified progression framework may provide added prognostic value. The descriptive inspection of visit-wise prediction errors suggested that the prognostic benefit of including atrophy stage tended to increase with longer follow-up duration. Finally, we demonstrate that this prognostic value can be capitalised to reduce the sample size needed for placebo-controlled, randomised clinical trials in SCA3. Excluding individuals with low-stage atrophy (e.g., atrophy stage below four) substantially reduced required sample sizes with minimal additional screen failures. These results are timely and relevant to inform trial design in SCA3, especially given the current emergence of first-in-human trials of ASO treatment. The optimal atrophy stage cut-off will depend on context-specific factors, including the cost of extra screening and the expected treatment effect. Atrophy stage could also be combined with other disease progression markers, such as fluid or digital biomarkers, to identify patients at the targeted disease stage. Notably, prognostic enrichment is just one potential application of atrophy staging in clinical trials, as it may also support sub-sample stratification, sample homogenisation, or predictive enrichment.

This study should be interpreted in light of its limitations. First, we were unable to fully assess and account for potential scanner effects. Although we did not observe statistically significant effects of scanner manufacturer or field strength on atrophy stage, our data showed trends suggesting that scanner setup may have some influence. Because SCA3 is a rare disease, assembling sufficiently large datasets requires pooling data across multiple scanners and sites. In our study, the mean was 9.2 baseline scans per site, with ten scanners each contributing only a single scan. This structure limits the applicability of harmonisation methods, such as ComBat, which require larger sample sizes per batch.^38^ Likewise, including scanner specifications in the *w*-scoring procedure was not feasible, as several scanner setups present in the SCA3 sample were absent in the healthy control group, where 95 out of 100 participants were scanned on Siemens 3 T systems. Future work should focus on developing standardisation procedures for the regional volumes used here, explicitly accounting for variability due to manufacturer, field strength, and potentially scanner model, software, and head coil. Second, a limitation of our study is that missing data were not imputed. As missingness was largely driven by study site, with some variables being entirely unavailable at certain sites, there were no within-site observations to inform plausible values. While this conservative approach reduced statistical power and potentially biased our results towards sites with more complete data, it avoids the risk of introducing spurious values. Importantly, in the longitudinal subset, missingness in predictor variables was minimal, making it unlikely that this limitation strongly affected our longitudinal analyses. Another limitation concerns the analysis of the relationship between ataxia severity and brain changes. Given the limited sensitivity of the SARA scale below a score of 3, such correlations should be interpreted with caution. Digital motion assessments may provide a more sensitive means of capturing subtle, early gait alterations.^20^^,^^39^^,^^40^ Finally, all assessments of out-of-sample generalisability in our study relied on internal cross-validation. The gold standard, however, is replication in an independent cohort, which should be addressed in future studies.

In conclusion, we present a purely data-driven model of brain atrophy progression in SCA3, tracing a clear and distinct sequence that originates in the caudal brainstem and strongly involves the cerebellar white matter. By mapping this sequence onto discrete atrophy stages, we establish a framework that not only captures present disease progression but also outperforms single-region volumetrics in predicting longitudinal ataxia progression. Notably, our data suggest that atrophy stages can be reliably determined in independent data and may contribute to more efficient clinical trial designs by enabling sample enrichment strategies that reduce required sample sizes with only modest increases in screen failure rates. Together, these results offer novel mechanistic insights into neurodegeneration in SCA3 with immediate translational relevance, supporting refined patient stratification and more efficient interventional studies.

## Contributors

H.B.: Methodology, Software, Validation, Formal analysis, Investigation, Data curation, Writing—Original Draft, Visualisation.; P.W., M.F., and T.S.: Formal analysis, Investigation, Resources, Writing—Review & Editing; M.C.F.J., T.J.R.R., A.R.M.M., Ho.J., Z.C., L.W., M.G.-E., B.K., D.Ö., B.v.d.W., J.v.G., A.D., G.C., M.S., L.S., P.G., H.G.-M., G.Ö., J.J., D.T., A.G.T., He.J., J.d.V., P.B., C.O., E.-M.R., J.D.S., K.R., J.I., J.H.-S., and D.K.: Resources, Writing—Review & Editing; T.K.: Writing—Review & Editing, Resources, Project administration, Funding acquisition; D.B.: Writing—Review & Editing, Supervision; J.F.: Data curation, Writing—Original Draft, Supervision, Project administration, Resources, Funding acquisition; DELCODE/DANCER study group and ESMI MRI study group: Resources, Writing—Review & Editing. All authors read and approved the final version of the manuscript. H.B., D.B., and J.F. accessed and verified the underlying data.

## Data sharing statement

Investigators may request access to deidentified data by contacting Jennifer Faber (Jennifer.Faber@dzne.de). Data sharing is contingent upon data transfer agreements between the requesting institution, the respective study sites, and the German Center for Neurodegenerative Diseases (DZNE). The SuStaIn algorithm is publicly available (https://github.com/ucl-pond/pySuStaIn). All methodological specifications necessary to reproduce the atrophy staging analyses are provided in this manuscript.

## Declaration of interests

H.B. declares payments from Elsevier (honoraria). T.J.R.R. declares payments from the São Paulo Research Foundation (FAPESP), Friedreich's Ataxia Research Alliance (FARA) (funding), Biogen (consulting fees) and PTC Therapeutics (consulting fees and honoraria). M.G.-E. declares payments from Ataxia UK, National Ataxia Foundation (NAF) (funding), Biogen (honoraria) and Aparito (travel support). B.v.d.W. declares payments from ZonMw, the Dutch Scientific Organization, Hersenstichting, and Christina Foundation (funding), Springer Nature (royalties), Biogen, Servier Laboratories, and Vico Therapeutics (consulting fees), Malaysia Movement Disorder Society (honoraria, travel support), and the Belgian Neurological Society (honoraria), received materials from Brugling Funds, and is an advisor for the International Parkinson and Movement Disorder Society and the Ataxia Global Initiative (AGI). A.D. declares payments from the French National Research Agency (ANR), Servier Laboratories, Wave Life Sciences, CHDI Foundation, Vico Therapeutics, PTC Therapeutics (funding), Biogen, UCB, FRM, and Huntix (consulting/advisory fees), partly holds patent B 06291873.5, is the president of the Société Francophone de Neurogénétique, and is the vice president of the scientific board of the Fondation pour la Recherche Medicale. M.S. declares payments from the Else Kröner Fresenius Stiftung (funding), UCB, Prevail Therapeutics, Ionis Pharmaceuticals, Orphazyme, Servier Laboratories, Reata Pharmaceuticals, AviadoBio, Gen-Orph, Biohaven, Zevra Therapeutics, Eli Lilly and Company, and Solaxa (consulting fees). L.S. declares payments from Servier Laboratories, UCB (funding), Reata Pharmaceuticals and Alexion Pharmaceuticals (consulting fees). H.G-M. declares payments from Ataxia UK (travel support). G.O. declares payments from the National Institutes of Health (NIH, U01NS104326, P41EB027061, P30NS076408, S10 OD017974, UL1TR000114), the NAF (funding), BrainSpec (licencing), Servier Laboratories, UCB, Lacerta Therapeutics (consulting fees), holds patent US 10,641,854 B2, and is an advisor for BrainSpec and the NAF. A.G.T. declares payments from the German Academic Exchange Service (travel support) and is stock owner of Novo Nordisk and Pfizer. C.O. declares payments from Alector, Transposon Therapeutics, and Denali Therapeutics (funding), Acadia Pharmaceuticals, Reata Pharmaceuticals, Otsuka Pharmaceutical, Eli Lilly and Company, Alexion Pharmaceuticals, Lykos Therapeutics, Zevra Therapeutics, Neuvivo, Sanofi, Biohaven, AviadoBio (consulting fees), and the Lewy Body Dementia Association (honoraria), is a member of the FTD Disorders Registry Scientific Advisory Board, the Tau Consortium Scientific Advisory Board, the AFTD Medical Advisory Council, and the International Society for Neurodegenerative Disease Scientific Advisory Board, and received materials from Alector, Transposon Therapeutics, and Denali Therapeutics. E.M.-R. declares payments from the NIH (R01 DA047088, R01 NS124065), the Baszucki Group Foundation (2023-005) (funding), and Aletheia (consulting fees), and is an advisor for BrainSpec. J.S. declares payments from the NAF, the ARSACS Foundation, the Once Upon a Time Foundation (funding), Oxford University Press, Elsevier, MacKeith Press, Springer (royalties), Biohaven (consulting fees), Harvard Medical School, NYU Medical School, University of Maryland, Barrow Neurological Institute, and Beth Israel Lahey Medical Center (honoraria), and McConnel Van Pelt (testimony), is a site PI for Biohaven, is the inventor of the Brief Ataxia Rating Scale, the Cerebellar Cognitive Affective/Schmahmann Syndrome Scale, the Patient Reported Outcome Measure of Ataxia, and the Cerebellar Neuropsychiatry Rating Scale versions 1 and 2, and is an advisor for the NAF. K.R. declares payments from the CHDI foundation, Roche, Novo Nordisk (funding), Eli Lilly and Company, and Eisai (funding, honoraria, consulting fees). J.I. declares payments from STADA (honoraria), Biogen, AbbVie, (honoraria, travel support) and Zambon (travel support). T.K. declares payments from the NIH (funding), Arrowhead Pharmaceuticals, Bristol-Myers Squibb, and UCB (consulting fees). D.B. is co-founder, stock holder, and part-time employee of neotiv GmbH. J.F. declares payments from the Advanced Clinician Scientist Programme (ACCENT, 01EO2107, German Federal Ministry of Research, Technology and Space [BMFTR]), the Ministry of Culture and Science of the State of North Rhine-Westphalia, the NAF (funding), Vico Therapeutics, Biogen (consulting fees, honoraria), the NAF, the FARA, and euro-ataxia (travel support), is an advisor for Vico Therapeutics, and is a member of the MDS Ataxia Study Group, a member of the Ataxia Global Initiative Steering Committee, and the lead of the Ataxia Global Initiative MR working group. The other authors report no competing interests related to this manuscript.

The ESMI MRI study group is part of ESMI, an EU Joint Programme–Neurodegenerative Disease Research (JPND) project (see www.jpnd.eu), supported through the following funding organisations under the aegis of JPND: Germany, Federal Ministry of Education and Research (BMBF; 01ED1602 A/B); Netherlands, The Netherlands Organisation for Health Research and Development; UK, Medical Research Council (MR/N028767/1). DELCODE and DANCER are funded by Clinical Research, German Center for Neurodegenerative Disorders (DZNE).

## Appendix A Supplementary data

Supplementary Figures and Tables

Study groups

Study Groups and Affiliations
