# Supplementary material for: Brain atrophy staging in spinocerebellar ataxia type 3 for clinical prognosis and trial enrichment
Source: eBioMedicine. 2025 Dec 23;123:106090. doi: 10.1016/j.ebiom.2025.106090 (PMC12800623; doi:10.1016/j.ebiom.2025.106090)
Supplement: Supplementary Figures and Tables [file mmc1.docx]

Supplementary Material

to the manuscript

*Brain atrophy staging in spinocerebellar ataxia type 3 for clinical prognosis and trial enrichment*

Baumeister et al.

# Supplementary methods

## Estimation of the age of onset

Age of onset was defined as the reported first occurrence of gait disturbances. For those reporting no gait difficulties, age of onset was estimated based on CAG repeat length, which was then subtracted from the current age.^1^

## SuStaIn modelling

SuStaIn was originally described by Young *et* al.^2^ Readers are advised to refer to this manuscript for a thorough methodological description of the SuStaIn model. The methods specific to the present SuStaIn model are described in this section.

As described in the main manuscript, regional volumes were first corrected for intracranial volume and were then *w*-scored in reference to the healthy control group, correcting for age and sex. The resulting values were then inverted, as SuStaIn assumes the modelled biomarkers to monotonously increase with disease progression. However, we report non-inverted *w*-scores for more intuitive understanding where lower *w*-scores reflect lower regional volumes. We then proceeded to define a SuStaIn model with twenty-five starting points for the expectation maximization procedure during model fitting and 10,000 Markov Chain Monte Carlo samples for uncertainty estimation. We repeated this procedure to identify up to *k* = 5 potential subtypes (i.e., distinct atrophy progression sequences) in the dataset.

Ten-fold cross-validation was employed to guide model selection (i.e., selecting the number of subtypes), and for assessing model robustness and generalizability. For this procedure, the dataset was randomly divided into ten folds. In each of ten iterations, a different fold was left out, and the SuStaIn model was trained on the remaining nine folds. The trained model was then used to classify the held-out fold. While the statistical procedure used to evaluate model generalizability from cross-validation results is described in the main manuscript, the following sections provide more details on model selection and sequence robustness testing.

### Model selection

Model selection followed the recommendations by Young *et al*. (ref.^2^). We evaluate both cross-validation information criterion (CVIC) and out-of-sample log-likelihood for selecting the model best fitting the training data where more complex model (a model with *k* subtypes) was preferred over a simpler model (a model with *k* − 1 subtypes) only if (i) CVIC*_k_* − CVIC*_k_*_−1_ < −3, and (ii) log-likelihood*_c_* − log-likelihood*_k_*_−1_ > 6.

### Sequence robustness testing

To assess the robustness of the identified atrophy progression sequence across data subsets, we calculated the statistical overlap (Bhattacharyya coefficient, *BC*) of the posterior distributions of event locations in the whole sequence across cross-validation folds. These values were then averaged to $\bar{BC}$. We repeated this procedure using randomly scrambled posterior distributions for each *w*-score threshold, yielding the reference value$\bar{{BC}_{0}}$.^3^

# References

1 Tezenas du Montcel S, Durr A, Rakowicz M, *et al.* Prediction of the age at onset in spinocerebellar ataxia type 1, 2, 3 and 6. *J Méd Genet* 2014; **51**: 479.

2 Young AL, Marinescu RV, Oxtoby NP, *et al.* Uncovering the Heterogeneity and Temporal Complexity of Neurodegenerative Diseases with Subtype and Stage Inference. *Nat Commun* 2018; **9**: 4273.

3 Oxtoby NP, Leyland L-A, Aksman LM, *et al.* Sequence of clinical and neurodegeneration events in Parkinson’s disease progression. *Brain* 2021; **144**: 975–88.

# Supplementary figures


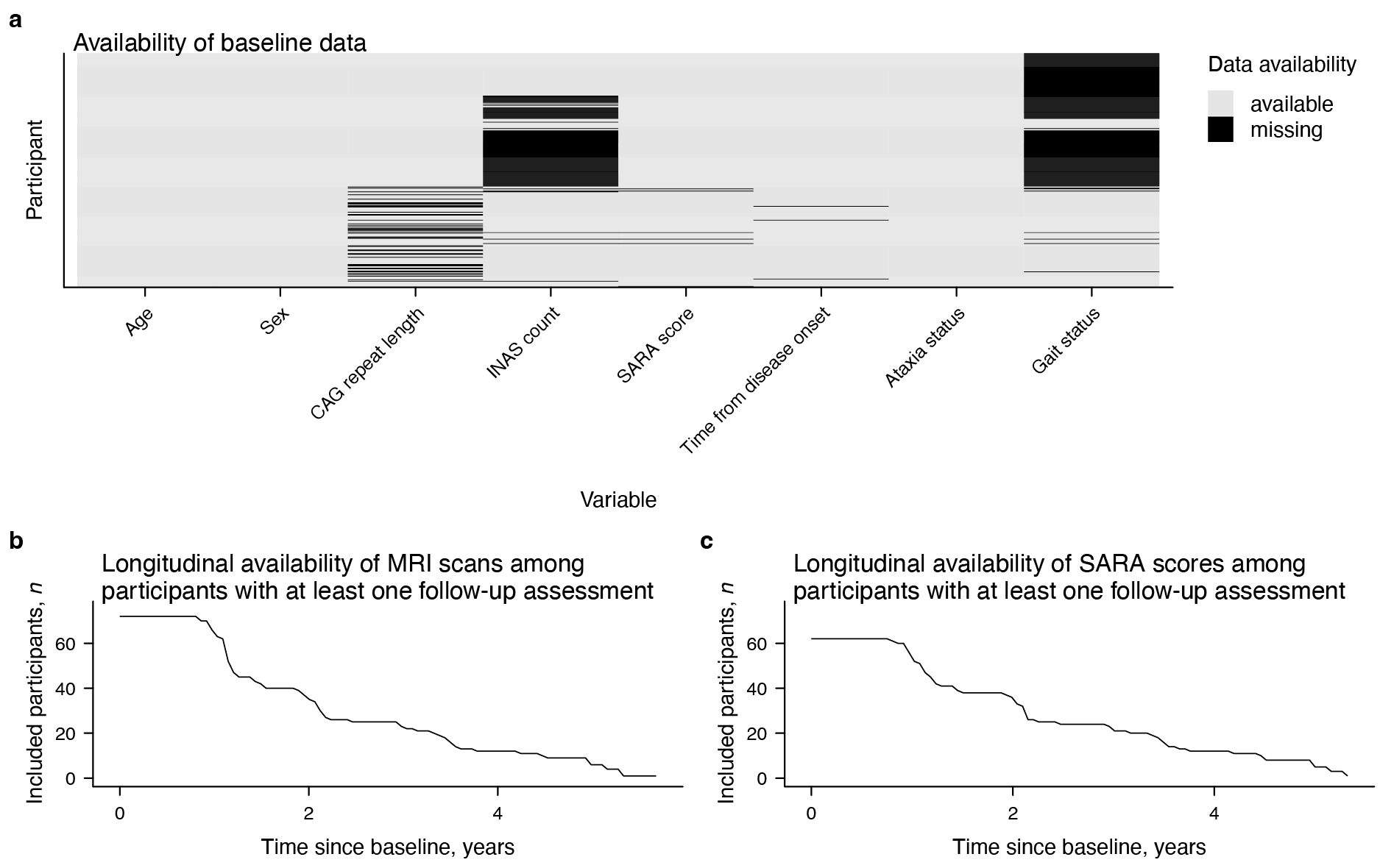


**Supplementary Figure 1 Overview of data availability.** (**a**) shows the availability of all analysed baseline variables while (**b**) and (**c**) show the availability of longitudinally recorded MRI scans and SARA scores, respectively. Note that for the plots in (**b**) and (**c**), only participants with at least two observations were considered.


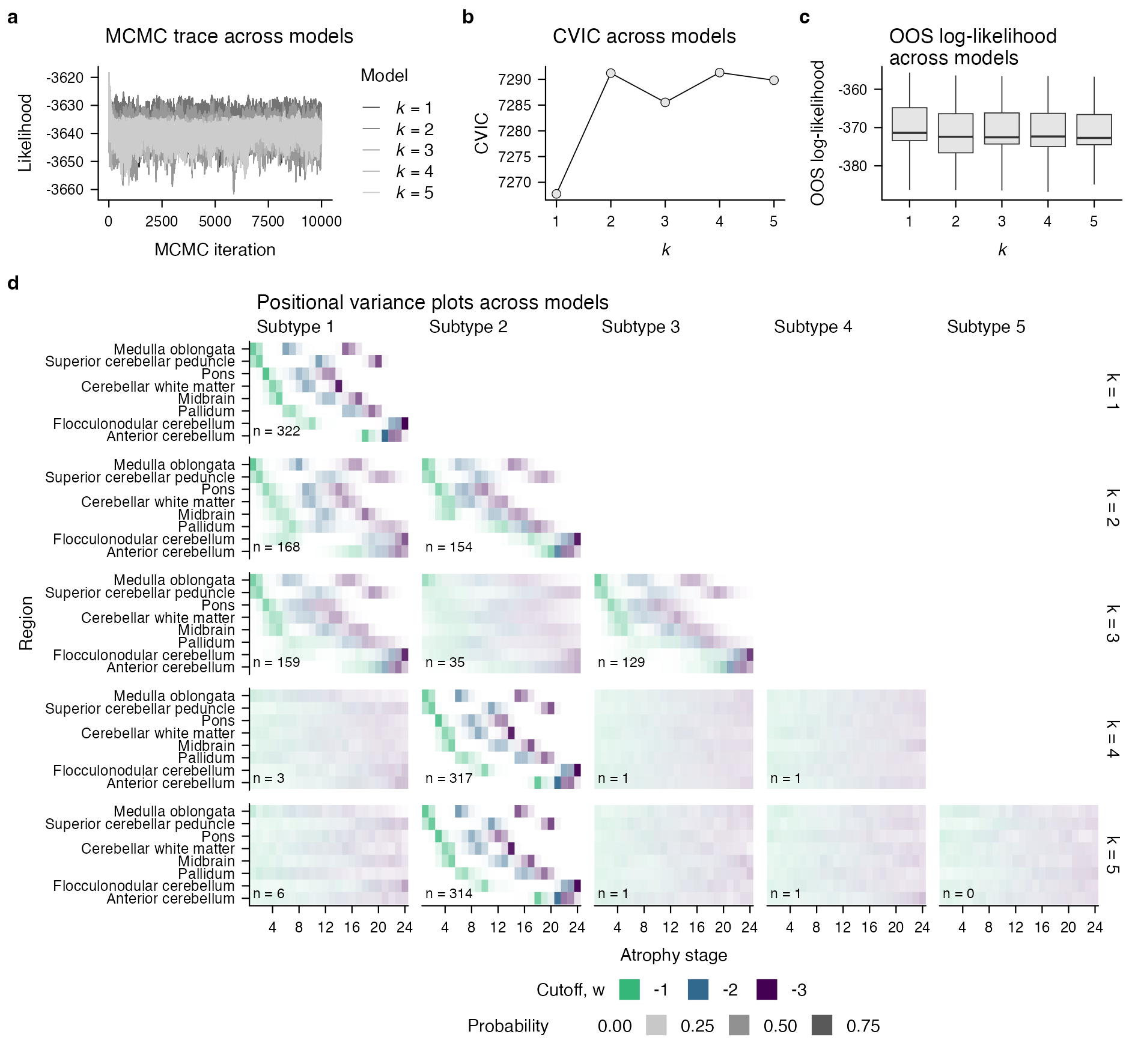


**Supplementary Figure 2 Ten-fold cross-validation indicates the presence of one distinct sequence of regional volume reduction in SCA3.** (**a**) Markov Chain Monte Carlo (MCMC) sampling was used to estimate the uncertainty of each *k*-subtype model. As traces of models estimating *k* > 1 subtypes are not clearly distinguished from the *k* = 1 solution, MCMC sampling shows no added benefit of modelling more than one atrophy progression sequence. (**b**) CVIC and (**c**) out-of-sample log-likelihoods across the fitted models indicate that a *k* = 1 subtype model best approximates the atrophy progression sequence in SCA3. (**d**) Positional variance plots visualize the atrophy progression sequence(s) across the fitted models. The sequence identified when setting *k* = 1 (one subtype) was replicated across models estimating *k* > 1 sequences. The additional sequences do not indicate alternative atrophy progression patterns. Abbreviations: OOS, out-of-sample.


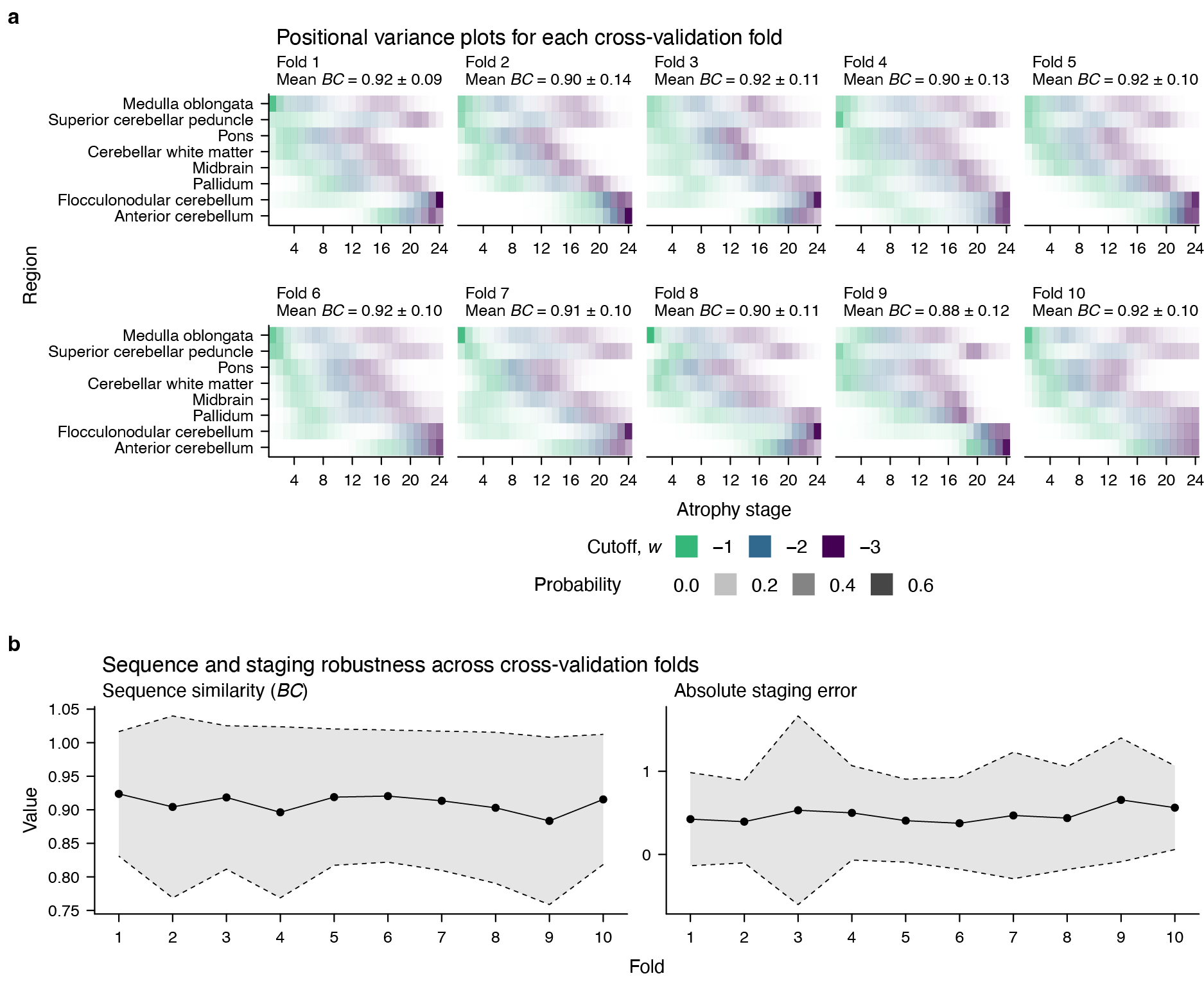


**Supplementary Figure 3 Model robustness results from ten-fold cross-validation.** (**a**) Positional variance plots visualize the atrophy sequences identified in each cross-validation iteration. Sequence overlap across iterations was quantified using Bhattacharyya coefficients (*BC*s), with mean ± *SD* values indicated in the plot headings and the left panel of (**b**). The right panel of (**b**) shows the mean and *SD* ribbons of absolute staging error per cross-validation fold, calculated from the absolute differences of atrophy stages derived from whole-sample model training and cross-validated out-of-sample predictions.


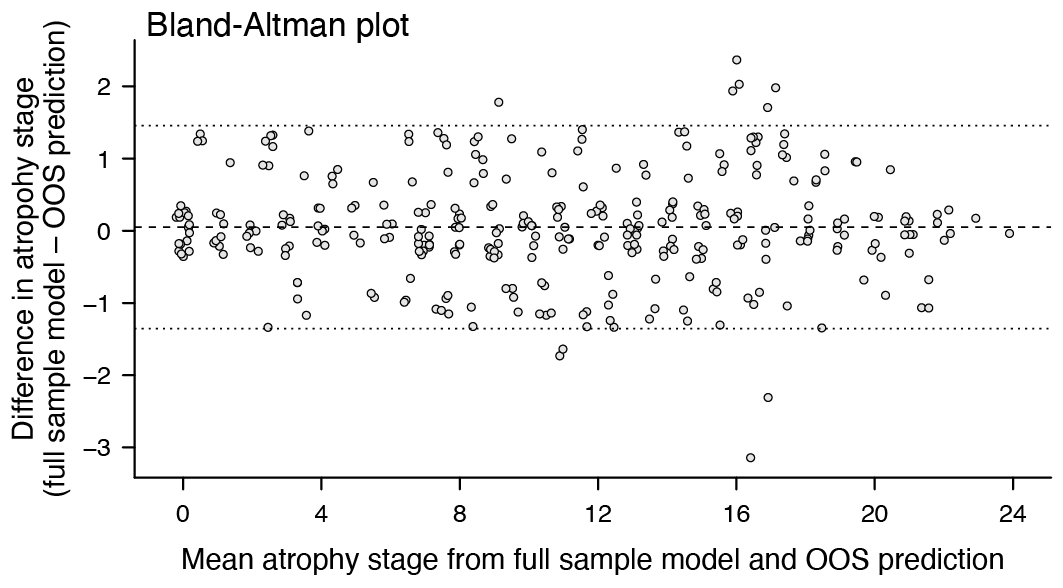


**Supplementary Figure 4 Bland-Altman plot of atrophy stages from whole-sample model training and out-of-sample (OOS) predictions.** This plot suggests similar robustness of classifications across atrophy stages. The dashed line represents the mean difference of atrophy stages from the full-sample model and cross-validated OOS predictions. The dotted lines mark the values at 1.96 *SD* of these differences. All atrophy stage data points are jittered by ± 0.5 along the respective axis to improve readability.


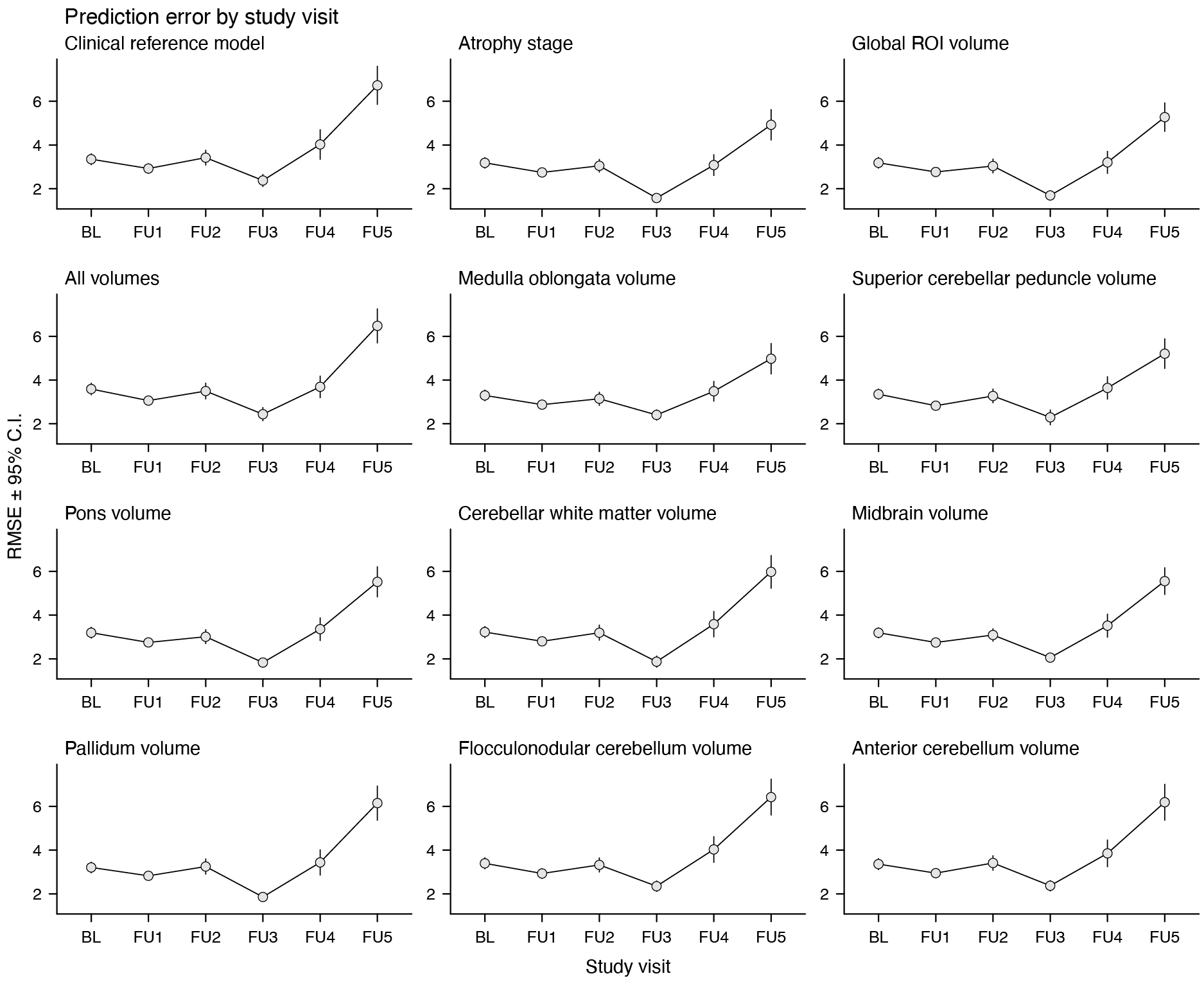


**Supplementary Figure 5** **Visit-wise prediction error for LMMs predicting longitudinal SARA trajectories, derived from cross-validation.** Models include the clinical reference model as well as models with different MRI readout added as predictors.

**
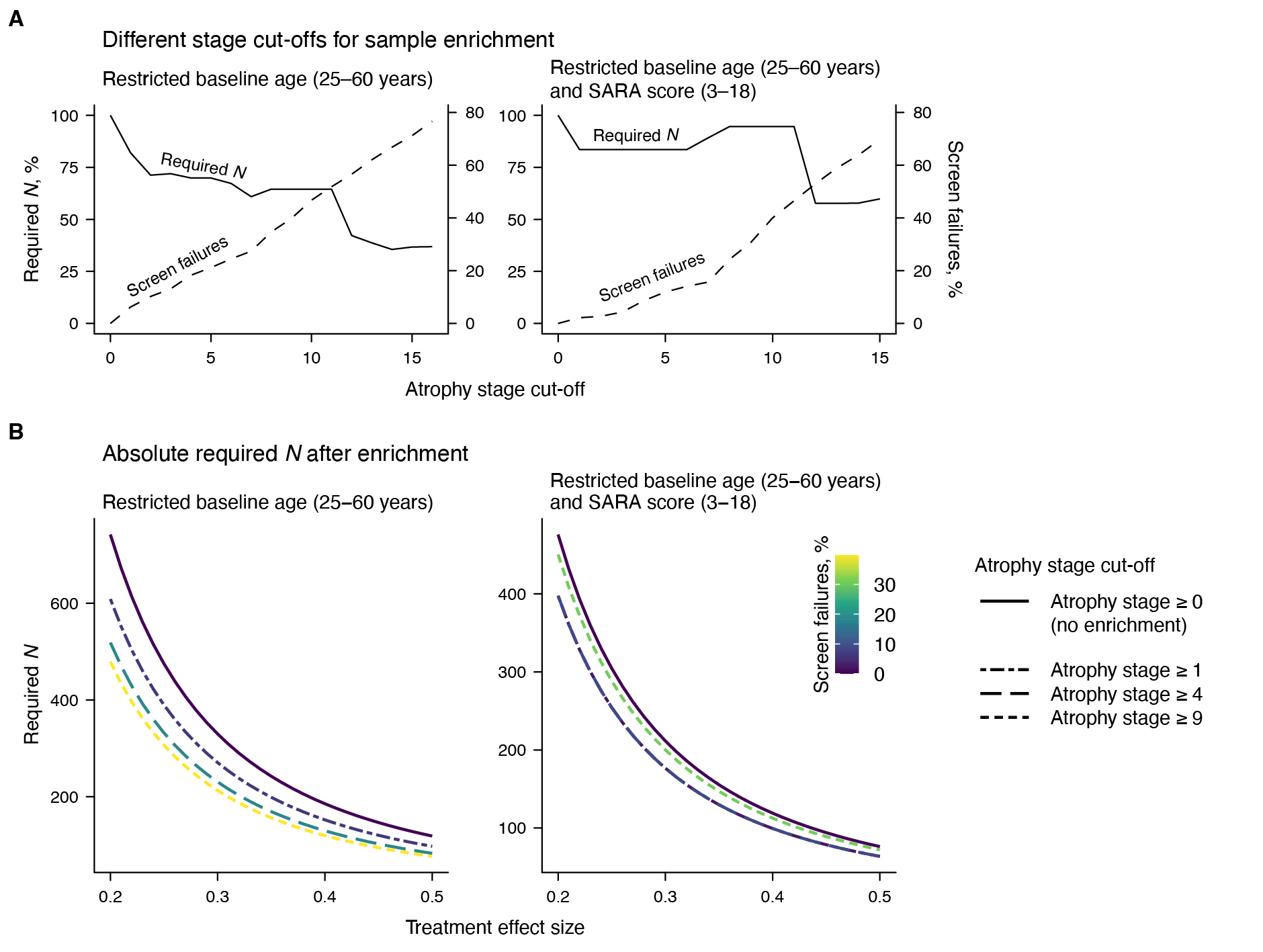
**

**Supplementary Figure 6 Sample size reductions for hypothetical two-year, placebo-controlled, parallel-group trial using atrophy stage as an enrichment variable along with eligibility criteria for age and SARA.** We simulated both trials with a limited age range (25–60 years; left column) and trials with limited age and SARA score (3–18) ranges (right column). (**A**) Expected reductions in sample size (*N*; left *y*-axis) and corresponding changes in screen failures (right *y*-axis) for cut-offs between atrophy stages 0 and 15/16 (right/left column) . Higher cut-offs were not modelled due to limited data availability. (**B**) Absolute required *N* for four enrichment scenarios: no enrichment (including all participants regardless of atrophy stage) and progressively excluding individuals below atrophy stages 1, 4, and 9. *N* represents the total number of participants across both study arms, assuming equal allocation per arm.

# Supplementary tables

**Supplementary Table 1** Overview of scanners and sequences.

| **Country** | **Site** | **Scanner vendor** | **Field strength, Tesla** | **TR, ms** | **TE, ms** | **TI, ms** | **Flip angle** | **Number of sclies** | **Field of view** | **Resolution, mm** | | | **Number of scans** | |
| --- | --- | --- | --- | --- | --- | --- | --- | --- | --- | --- | --- | --- | --- | --- |
|  |  |  |  |  |  |  |  |  |  | **x** | **y** | **z** | **At base-line** | **In total** |
| Brazil | Campinas | Phillips, Achieva | 3 | 7 | 3201 | *NA* | 8 | 180 | 240 x 240 | 1 | 1 | 1 | 53 | 53 |
| China | Changsha | SIEMENS, Prisma | 3 | 2300 | 2,98 | 900 | 9 | 192 | 256 x 256 | 0,9 | 0,94 | 0,94 | 50 | 50 |
| France | Paris | GE, SIGNA HDX | 3 | 5,96 | 2,532 | 380 | 15 | 134 | 512 x 512 | 0,89 | 0,89 | 1 | 2 | 2 |
|  |  | GE, Optima MR360 | 1,5 | 7,94 | 4,2 | *NA* | 15 | 232 | 512 x 512 | 0,5 | 0,5 | 1,4 | 1 | 1 |
|  |  | GE, SIGNA HDX | 3 | 6,61 | 2,828 | *NA* | 15 | 158 | 256 x 256 | 1 | 1 | 1,2 | 1 | 1 |
|  |  | GE, Optima MR450w | 1,5 | 9,28 | 4,2 | *NA* | 15 | 252 | 512 x 512 | 0,5 | 0,5 | 1,2 | 1 | 1 |
|  |  | SIEMENS, TRIO | 3 | 2300 | 2,98 | 900 | 9 | 160 | 256 x 240 | 1 | 1 | 1,1 | 19 | 19 |
| Germany | Aachen | SIEMENS, Prisma | 3 | 2500 | 4,37 | 1100 | 7 | 192 | 256 x 256 | 1 | 1 | 1 | 5 | 7 |
|  | Berlin | SIEMENS, TimTrio | 3 | 2500 | 4,33 | 1100 | 7 | 192 | 256 x 256 | 1 | 1 | 1 | 6 | 6 |
|  |  | SIEMENS, Prisma fit | 3 | 2500 | 4,37 | 1100 | 7 | 192 | 256 x 256 | 1 | 1 | 1 | 9 | 9 |
|  | Bonn | SIEMENS, Skyra | 3 | 2500 | 4,37 | 1100 | 7 | 192 | 256 x 256 | 1 | 1 | 1 | 85 | 148 |
|  |  | Philips, Intera | 1,5 | 15,31 | 3,6 | *NA* | 30 | 140 | 256 x 256 | 1 | 1 | 1 | 2 | 2 |
|  |  | Philips, Intera | 3 | 8,29 | 3,8 | *NA* | 8 | 140 | 256 x 256 | 1 | 1 | 1 | 8 | 8 |
|  | Essen | SIEMENS, Biograph | 3 | 2500 | 4,37 | 1100 | 7 | 192 | 256 x 256 | 1 | 1 | 1 | 13 | 37 |
|  | Frankfurt | SIEMENS, Verio | 3 | 1500 | 2,93 | 900 | 9 | 160 | 512 x 512 | 0,5 | 0,5 | 1 | 9 | 9 |
|  | Heidelberg | SIEMENS, TRIO | 3 | 2501 | 4,33 | 1100 | 7 | 192 | 256 x 256 | 1 | 1 | 1 | 11 | 11 |
|  | Munich | SIEMENS, Skyra | 3 | 2500 | 4,37 | 1100 | 7 | 192 | 256 x 256 | 1 | 1 | 1 | 1 | 1 |
|  | Rostock | SIEMENS, Skyra fit | 3 | 2500 | 4,37 | 1100 | 7 | 192 | 256 x 256 | 1 | 1 | 1 | 6 | 6 |
|  | Tübingen | SIEMENS, Sonata | 1,5 | 20 | 5 | *NA* | 30 | 160 | 256 x 256 | 0,9 | 0,9 | 0,9 | 1 | 1 |
|  |  | SIEMENS, Avanto | 1,5 | 1900 | 3,93 | 1100 | 15 | 160 | 256 x 256 | 1 | 1 | 1 | 11 | 11 |
|  |  | SIEMENS, Skyra | 3 | 2300 | 23,2 | 900 | 8 | 192 | 256 x 256 | 0,9 | 0,9 | 0,9 | 1 | 1 |
|  |  | SIEMENS, Sonata | 1,5 | 1150 | 32,2 | *NA* | 15 | 160 | 512 x 512 | 0,48 | 0,48 | 1 | 1 | 1 |
|  |  | SIEMENS, Sonata | 1,5 | 1430 | 36,8 | *NA* | 15 | 176 | 512 x 512 | 0,5 | 0,5 | 1 | 1 | 1 |
|  |  | SIEMENS, Avanto | 1,5 | 1900 | 30,9 | 1100 | 15 | 160 | 256 x 256 | 1 | 1 | 1 | 1 | 1 |
|  |  | SIEMENS, Skyra | 3 | 2300 | 11720 | 900 | 8 | 192 | 256 x 156 | 0,9 | 0,9 | 0,9 | 13 | 13 |
| Spain | Santander | Philips, Achieva | 3 | 15 | 5 | *NA* | 8 | 160 | 256 x 256 | 0,9 | 0,9 | 1 | 2 | 2 |
| The Netherlands | Groningen | SIEMENS, Prisma | 3 | 2500 | 4,37 | 1100 | 7 | 192 | 256 x 256 | 1 | 1 | 1 | 10 | 12 |
|  | Nijmegen | SIEMENS, Skyra | 3 | 2500 | 4,37 | 1100 | 7 | 192 | 256 x 256 | 1 | 1 | 1 | 21 | 54 |
|  |  | SIEMENS, Avanto | 1,5 | 1900 | 2,55 | 1100 | 15 | 176 | 256 x 256 | 1 | 1 | 1 | 1 | 1 |
|  |  | SIEMENS, Avanto | 1,5 | 1900 | 2,55 | 1100 | 15 | 176 | 256 x 256 | 0,9 | 0,9 | 1 | 1 | 1 |
|  |  | SIEMENS, Trio | 3 | 2300 | 3,03 | 1100 | 8 | 192 | 256 x 256 | 1 | 1 | 1 | 24 | 24 |
| UK | London | SIEMENS, Prisma | 3 | 2500 | 4,37 | 1100 | 7 | 192 | 256 x 256 | 1 | 1 | 1 | 23 | 41 |
| US | Baltimore | SIEMENS, Prisma fit | 3 | 2500 | 4,37 | 1100 | 7 | 192 | 256 x 256 | 1 | 1 | 1 | 8 | 8 |
|  | Boston | SIEMENS, TrioTim | 3 | 2500 | 4,33 | 1100 | 7 | 192 | 256 x 256 | 1 | 1 | 1 | 3 | 3 |
|  | Minnesota | SIEMENS, Prisma Fit | 3 | 2500 | 4,37 | 1100 | 7 | 192 | 256 x 256 | 1 | 1 | 1 | 18 | 18 |

Abbreviations: NA, missing data. TR, repetition time. TE, echo time. TI, inversion time

**Supplementary Table 2** Slope coefficients used for adjusting regional volumes for intracranial volume.

| **Regional volume** | **Slope** |
| --- | --- |
| Medulla oblongata | 0.0024 |
| Superior cerebellar peduncle | 0.0002 |
| Pons | 0.0094 |
| Cerebellar white matter | 0.0065 |
| Midbrain | 0.0038 |
| Pallidum | 0.0011 |
| Flocculonodular cerebellum | 0.0002 |
| Anterior cerebellum | 0.0037 |

Regional volumes were adjusted for intracranial volume (ICV) using the formula volume_corr._ = volume_raw_ – slope ⨉ (ICV – ICV_mean_), where ICV_mean_ = 1577991.86.

**Supplementary Table 3** Summary statistics of linear regression models used for *w*-scoring regional volumetric atrophy markers.

| **Predicted marker^a^** | ***SD*_resid._** | **Predictor** | ***b*** | ***SE*** | ***t*** | ***p*** |
| --- | --- | --- | --- | --- | --- | --- |
| Medulla oblongata | 411.10 | Intercept | 4912.66 | 141.39 | 34.75 | < .0001**** |
|  |  | Age, years | –2.89 | 3.03 | –0.95 | .3428 |
|  |  | Sex, female | 55.02 | 83.53 | 0.66 | .5117 |
| Superior cerebellar peduncle | 51.57 | Intercept | 224.93 | 17.74 | 12.68 | < .0001**** |
|  |  | Age, years | 1.06 | 0.38 | 2.79 | .0063** |
|  |  | Sex, female | 4.89 | 10.48 | 0.47 | .6417 |
| Pons | 1477.98 | Intercept | 14777.20 | 508.32 | 29.07 | < .0001**** |
|  |  | Age, years | 2.78 | 10.89 | 0.26 | .7992 |
|  |  | Sex, female | 154.47 | 300.32 | 0.51 | .6082 |
| Cerebellar white matter | 1185.25 | Intercept | 13777.18 | 407.64 | 33.80 | < .0001**** |
|  |  | Age, years | –18.38 | 8.73 | –2.11 | .0379* |
|  |  | Sex, female | 242.70 | 240.84 | 1.01 | .3161 |
| Midbrain | 486.56 | Intercept | 6532.67 | 167.34 | 39.04 | < .0001**** |
|  |  | Age, years | –0.02 | 3.58 | 0.00 | .9962 |
|  |  | Sex, female | 20.50 | 98.87 | 0.21 | .8362 |
| Pallidum | 169.05 | Intercept | 2316.60 | 58.14 | 39.84 | < .0001**** |
|  |  | Age, years | –6.10 | 1.25 | –4.90 | < .0001**** |
|  |  | Sex, female | 22.76 | 34.35 | 0.66 | .5091 |
| Flocculonodular cerebellum | 78.92 | Intercept | 683.66 | 27.14 | 25.19 | < .0001**** |
|  |  | Age, years | –3.49 | 0.58 | –6.00 | < .0001**** |
|  |  | Sex, female | 8.86 | 16.04 | 0.55 | .5821 |
| Anterior cerebellum | 620.19 | Intercept | 7067.82 | 213.30 | 33.14 | < .0001**** |
|  |  | Age, years | –12.41 | 4.57 | –2.72 | .0078** |
|  |  | Sex, female | –145.17 | 126.02 | –1.15 | .2522 |

Models were fitted to provide predicted volumes (volume_predicted_) for *w*-score calculation, which followed the formula: volume*_w_*= (volume_corr._ – volume_predicted_) / *SD*_resid._. Note that for SuStaIn modelling, these *w*-scores were inverted so that higher values corresponded to greater atrophy. volume_corr_ refers to volumes after adjustment for intracranial volume. **p* < .05. ***p* < .01. *****p* < .0001

**Supplementary Table 4** Summary statistics of linear regression models predicting atrophy stage.

| **Predictor** | ***b*** | ***SE*** | ***t*** | ***p*** |
| --- | --- | --- | --- | --- |
| Intercept | 14.52 | 2.42 | 5.99 | < .0001**** |
| Time from disease onset, years | 0.36 | 0.03 | 11.29 | < .0001**** |
| Manufacturer, Philips | –4.28 | 2.42 | –1.77 | .0774 |
| Manufacturer, Siemens | –3.73 | 2.34 | –1.59 | .1120 |
| Field strength, 3T | –2.20 | 1.20 | –1.83 | .0686 |

**** *p* < .0001.

**Supplementary Table 5** Summary statistics of linear mixed effects models predicting longitudinal atrophy stages.

| **Predictor** | ***b*** | **95% C.I.** | ***SE*** | **df** | ***t*** | ***p*** |
| --- | --- | --- | --- | --- | --- | --- |
| ***Time since baseline only model: R^2^_conditional_ = .99, R^2^_marginal_ = .01*** | | | | | | |
| Intercept | 12.05 | [10.52; 13.58] | 0.78 | 71.06 | 15.52 | < .0001**** |
| Time since baseline, years | 0.49 | [ 0.32; 0.66] | 0.09 | 53.17 | 5.68 | < .0001**** |
| ***Time since baseline and ataxia status model: R^2^_conditional_ = .99, R^2^_marginal_ = .49*** | | | | | | |
| Intercept | 14.73 | [13.43; 16.03] | 0.67 | 70.17 | 22.14 | < .0001**** |
| Time since baseline, years | 0.59 | [0.40; 0.79] | 0.10 | 52.38 | 6.00 | < .0001**** |
| Baseline ataxia status, ataxic | –10.12 | [13.43; 16.03] | 1.30 | 70.23 | –7.82 | < .0001**** |
| Time since baseline, years ⨉ baseline ataxia status, ataxic | –0.40 | [0.40; 0.79] | 0.20 | 61.15 | –2.04 | .0461* |

All models included participant-wise random slopes and intercepts. **p* < .05. *****p* < .0001. Abbreviations: *SE*, standard error.

**Supplementary Table 6** Estimated marginal means of the time-dependent linear trend in atrophy stages, stratified by baseline ataxia status.

| **Baseline ataxia status** | **Estimated marginal mean** | **95% C.I.** | ***SE*** | **df** | ***t*** | ***p*** |
| --- | --- | --- | --- | --- | --- | --- |
| Ataxic | 0.59 | [0.39; 0.79] | 0.10 | 51.20 | 5.92 | < .0001**** |
| Preataxic | 0.19 | [–0.15; 0.53] | 0.17 | 63.20 | 1.12 | .2666 |

All models included participant-wise random slopes and intercepts. All estimates are corrected for age and sex. **p* < .05. Abbreviations: *SE*, standard error.

**Supplementary Table 7** Summary statistics of an ANCOVA model predicting baseline atrophy stages.

| **Predictor** | **Sum of squares** | **df** | **Mean squares** | ***F*** | ***p*** | ***η*^2^_partial_** | **95% C.I.** |
| --- | --- | --- | --- | --- | --- | --- | --- |
| Age | 553.79 | 1 | 553.79 | 23.02 | < .0001**** | 0.14 | [0.06; 1.00] |
| Sex, female | 23.08 | 1 | 23.08 | 0.96 | .3291 | 0.01 | [0.00; 1.00] |
| Gait status | 1907.81 | 3 | 635.94 | 26.43 | < .0001**** | 0.36 | [0.25; 1.00] |
| Residuals | 3392.07 | 141 | 24.06 |  |  |  |  |

*****p* < .0001.

**Supplementary Table 8** Summary statistics *post hoc* comparisons of gait status groups in baseline atrophy stage.

| **Contrast** | ***b*** | **95% C.I.** | ***SE*** | ***t*** | ***p*_FDR_** |
| --- | --- | --- | --- | --- | --- |
| gait disturbance *versus* normal gait | 8.17 | [4.84; 11.49] | 1.30 | 6.30 | < .0001**** |
| walking aid *versus* normal gait | 12.56 | [8.84; 16.28] | 1.45 | 8.65 | < .0001**** |
| wheelchair *versus* normal gait | 13.34 | [7.23; 19.45] | 2.38 | 5.60 | < .0001**** |
| walking aid *versus* gait disturbance | 4.39 | [1.82; 6.96] | 1.00 | 4.38 | < .0001**** |
| wheelchair *versus* gait disturbance | 5.17 | [–0.17; 10.51] | 2.08 | 2.48 | .0171* |
| wheelchair *versus* walking aid | 0.78 | [–4.81; 6.37] | 2.18 | 0.36 | .7211 |

All estimates are corrected for age and sex. **p* < .05. *****p* < .0001. Abbreviations: *SE*, standard error.

**Supplementary Table 9** Summary statistics of multiple regression models used to estimate the baseline associations of atrophy stage.

| **Parameter** | ***b*** | **95% C.I.** | ***t*** | **df** | ***p*** | **Standardised *β*** | **95% C.I.** |
| --- | --- | --- | --- | --- | --- | --- | --- |
| ***Outcome: Atrophy stage (R^2^_adjusted_ = 0.30)*** | | | | | | | |
| Intercept | 8.99 | [6.52; 11.46] | 7.15 | 315.00 | < .0001**** | 0.00 | [–0.09; 0.09] |
| Age, years | 0.00 | [–0.06; 0.05] | –0.10 | 315.00 | .9168 | –0.01 | [–0.11; 0.10] |
| Sex, female | –0.43 | [–1.57; 0.71] | –0.74 | 315.00 | .4592 | –0.03 | [–0.13; 0.06] |
| Time from onset, years | 0.37 | [0.29; 0.44] | 9.99 | 315.00 | < .0001**** | 0.55 | [0.44; 0.66] |
| ***Outcome: SARA score (R^2^_adjusted_ = 0.38)*** | | | | | | | |
| Intercept | –1.83 | [–4.27; 0.61] | –1.48 | 312.00 | .1410 | 0.00 | [–0.09; 0.09] |
| Age, years | 0.12 | [0.07; 0.17] | 4.56 | 312.00 | < .0001**** | 0.21 | [0.12; 0.30] |
| Sex, female | 0.49 | [–0.69; 1.67] | 0.82 | 312.00 | .4127 | 0.04 | [–0.05; 0.12] |
| Atrophy stage | 0.58 | [0.48; 0.68] | 11.52 | 312.00 | < .0001**** | 0.53 | [0.44; 0.62] |
| ***Outcome: INAS score (R^2^_adjusted_ = 0.38)*** | | | | | | | |
| Intercept | 1.50 | [0.25; 2.74] | 2.38 | 204.00 | .0184* | 0.00 | [–0.13; 0.13] |
| Age, years | 0.03 | [0.00; 0.06] | 2.05 | 204.00 | .0420* | 0.14 | [0.01; 0.28] |
| Sex, female | –0.18 | [–0.77; 0.41] | –0.59 | 204.00 | .5582 | –0.04 | [–0.16; 0.09] |
| Atrophy stage | 0.12 | [0.07; 0.17] | 4.63 | 204.00 | < .0001**** | 0.32 | [0.18; 0.45] |

**p* < .05. *****p* < .0001.

**Supplementary Table 10** Estimated marginal means of the atrophy status-dependent linear trend in time from onset, stratified by baseline ataxia status.

| **Ataxia status** | **EMM** | **95% C.I.** | ***SE*** | **df** | ***t*** | ***p*** | **Standardised EMM** | **95% C.I.** |
| --- | --- | --- | --- | --- | --- | --- | --- | --- |
| Preataxic | 0.21 | [0.07; 0.36] | 0.07 | 313.00 | 2.93 | .0037** | 0.32 | [0.10; 0.53] |
| Ataxic | 0.27 | [0.15; 0.39] | 0.06 | 313.00 | 4.30 | < .0001**** | 0.40 | [0.22; 0.58] |

All estimates are corrected for age and sex. ***p* < .01. *****p* < .0001. Abbreviations: EMM, estimated marginal mean. *SE*, standard error.

**Supplementary Table 11** Summary statistics of multiple regression models used to estimate the baseline associations of atrophy stage with SARA in preataxic and ataxic SCA3 mutation carriers.

| **Parameter** | ***b*** | **95% C.I.** | ***t*** | **df** | ***p*** | **Standardised *β*** | **95% C.I.** |
| --- | --- | --- | --- | --- | --- | --- | --- |
| ***Model in preataxic SCA3 mutation carriers (R^2^_adjusted_ = 0.19)*** | | | | | | | |
| Intercept | –0.08 | [–1.00; 0.85] | –0.17 | 62.00 | .8686 | 0.00 | [–0.21; 0.21] |
| Age, years | 0.03 | [0.00; 0.05] | 2.30 | 62.00 | .0248* | 0.26 | [0.03; 0.48] |
| Sex, female | –0.08 | [–0.54; 0.37] | –0.36 | 62.00 | .7173 | –0.04 | [–0.26; 0.18] |
| Atrophy stage | 0.09 | [0.05; 0.14] | 3.86 | 62.00 | 0.0003 | 0.43 | [0.21; 0.65] |
| ***Model in ataxic SCA3 mutation carriers (R^2^_adjusted_ = 0.24)*** | | | | | | | |
| Intercept | 3.56 | [0.59; 6.54] | 2.36 | 246.00 | .0191* | 0.00 | [–0.11; 0.11] |
| Age, years | 0.07 | [0.01; 0.12] | 2.39 | 246.00 | .0174* | 0.14 | [0.02; 0.25] |
| Sex, female | 1.49 | [0.21; 2.77] | 2.29 | 246.00 | .0226* | 0.13 | [0.02; 0.24] |
| Atrophy stage | 0.40 | [0.28; 0.52] | 6.73 | 246.00 | < .0001**** | 0.39 | [0.27; 0.50] |

**p* < .05. *****p* < .0001.

**Supplementary Table 12** Estimated marginal means of the INAS count-dependent linear trend in atrophy stage, stratified by baseline ataxia status.

| **Ataxia status** | **EMM** | **95% C.I.** | ***SE*** | **df** | ***t*** | ***p*** | **Standardised EMM** | **95% C.I.** |
| --- | --- | --- | --- | --- | --- | --- | --- | --- |
| Preataxic | 0.04 | [–0.09; 0.17] | 0.07 | 202.00 | 0.64 | .5206 | 0.26 | [–0.54; 1.06] |
| Ataxic | 0.05 | [0.00; 0.11] | 0.03 | 202.00 | 1.96 | .0513 | 0.33 | [0.00; 0.66] |

All estimates are corrected for age and sex. Abbreviations: EMM, estimated marginal mean. *SE*, standard error.

**Supplementary Table 13** Estimates of atrophy stage ⨉ time since baseline effects on longitudinal SARA scores, estimated using linear mixed effects models.

| **Atrophy marker, *w*-scored** | **Global model statistics** | | | **Statistics for the atrophy marker ⨉ time since baseline (years) effect** | | | | | |
| --- | --- | --- | --- | --- | --- | --- | --- | --- | --- |
|  | **R^2^_conditional_** | **R^2^_marginal_** | **AIC** | ***b*** | **95% C.I.** | ***SE*** | **df** | ***t*** | ***p*** |
| Atrophy stage | 0.96 | 0.83 | 223.36 | 0.08 | 4 | 0.03 | 31.34 | 2.53 | .0168* |
| Medulla volume | 0.96 | 0.82 | 229.05 | 0.07 | [0.03; 0.11] | 0.02 | 27.92 | 2.88 | .0075** |
| Superior cerebellar peduncle volume | 0.96 | 0.82 | 228.65 | 0.07 | [0.01; 0.12] | 0.03 | 25.64 | 2.28 | .0314* |
| Pons volume | 0.96 | 0.83 | 225.59 | 0.06 | [0.00; 0.12] | 0.03 | 36.52 | 1.85 | .0725 |
| Cerebellar white matter | 0.96 | 0.82 | 230.63 | 0.04 | [–0.02; 0.10] | 0.03 | 43.56 | 1.18 | .2438 |
| Midbrain volume | 0.96 | 0.83 | 227.23 | 0.05 | [0.00; 0.10] | 0.03 | 30.17 | 1.74 | .0925 |
| Pallidum volume | 0.96 | 0.81 | 233.85 | 0.03 | [–0.02; 0.08] | 0.03 | 32.01 | 0.91 | .3686 |
| Flocculonodular cerebellum volume | 0.96 | 0.80 | 237.14 | 0.05 | [0.01; 0.09] | 0.02 | 35.96 | 2.00 | .0531 |
| Anterior cerebellum volume | 0.96 | 0.80 | 238.86 | 0.03 | [–0.01; 0.08] | 0.02 | 44.24 | 1.41 | .1664 |
| Global ROI volume | 0.96 | 0.83 | 226.16 | 0.06 | [0.01; 0.12] | 0.03 | 35.02 | 2.03 | .0500 |

All models included participant-wise random slopes and intercepts. All models included age, sex, length of the CAG expanded allele, baseline gait status, and baseline INAS count as well as their interactions with time since baseline as covariates. **p* < .05. ***p* < .01. Abbreviations: *SE*, standard error.

**Supplementary Table 14** Cross-validated prediction error (RMSE) for each LMM including a different baseline MRI readout as well as test statistics for *t*-tests comparing RMSEs with the clinical reference model (RMSE = 3.85 [3.59; 4.10]).

| **Included atrophy marker** | **RMSE** | **95% C.I.** | ***t*** | ***p*_raw_** | ***p*_FDR_** |
| --- | --- | --- | --- | --- | --- |
| All volumes | 3.91 | [3.67; 4.15] | –0.19 | .8465 | .8465 |
| Anterior cerebellum volume | 3.77 | [3.53; 4.01] | 0.83 | .4105 | .5017 |
| Atrophy stage | 3.25 | [3.04; 3.46] | 2.08 | .0398* | .2063 |
| Cerebellar white matter volume | 3.57 | [3.32; 3.81] | 1.30 | .1974 | .3102 |
| Flocculonodular cerebellum volume | 3.79 | [3.57; 4.02] | 0.37 | .7117 | .7829 |
| Global ROI volume | 3.34 | [3.12; 3.56] | 1.97 | .0521 | .2063 |
| Medulla oblongata volume | 3.48 | [3.27; 3.69] | 1.38 | .1702 | .3102 |
| Midbrain volume | 3.39 | [3.18; 3.59] | 1.80 | .0750 | .2063 |
| Pallidum volume | 3.60 | [3.36; 3.85] | 1.31 | .1940 | .3102 |
| Pons volume | 3.35 | [3.13; 3.58] | 1.83 | .0705 | .2063 |
| Superior cerebellar peduncle volume | 3.49 | [3.26; 3.71] | 1.21 | .2278 | .3132 |

**p* < .05.
