# Supplementary material for: Brain atrophy staging in spinocerebellar ataxia type 3 for clinical prognosis and trial enrichment
Source: eBioMedicine. 2025 Dec 23;123:106090. doi: 10.1016/j.ebiom.2025.106090 (PMC12800623; doi:10.1016/j.ebiom.2025.106090)
Supplement: Study groups [file mmc2.docx]

**ESMI MRI study group**

| **First names** | **Surnames** |
| --- | --- |
| Kennet | Teichmann |
| Sarah | Bernsen |
| Katharina | Hill |
| Ilse | Willemse |
| Teije | van Prooije |
| Friedrich | Erdlenbruch |
| Thomas | Ernst |
| Benjamin | Bender |
| Johann E. | Jende |
| Khalaf | Bushara |
| Leire | Manrique |
| Pauline | Lallemant-Dudek |
| Sandro | Romanzetti |
| Alexander | Lange |
| Maya | Shrestha |
| Anton | Ludwig |
| Alena | Rosenow |
| Tim | Elter |
| Magda M. | Santana |
| Eberhard | Pracht |
| Tony | Stoecker |

**DELCODE/DANCER study group**

| **First names** | **Surnames** |
| --- | --- |
| Falk | Lüsebrink |
| Stefan | Hetzer |
| Michael | Ewers |
| Julian | Hellmann-Regen |
| Eike | Spruth |
| Daniel | Janowitz |
| Ingo | Kilimann |
| Marie T. | Kronmüller |
| Annika | Spottke |
| Oliver | Peters |
| Josef | Priller |
| Katharina | Buerger |
| Stefan | Teipel |
| Frank | Jessen |
| Emrah | Düzel |
| Anna | Gamez |
| Hannah | Asperger |
| Okka | Kimmich |
| Gabor C. | Petzold |
