# Supplementary material for: Brain atrophy staging in spinocerebellar ataxia type 3 for clinical prognosis and trial enrichment
Source: eBioMedicine. 2025 Dec 23;123:106090. doi: 10.1016/j.ebiom.2025.106090 (PMC12800623; doi:10.1016/j.ebiom.2025.106090)
Supplement: Study Groups and Affiliations [file mmc3.docx]

# ESMI MRI study group

Kennet Teichmann^1^, Sarah Bernsen^1,2^, Katharina Hill^1,2^, Ilse Willemse^3^, Teije van Prooije^3^, Friedrich Erdlenbruch^4^, Thomas M. Ernst^4^, Benjamin Bender^5^, Johann M. E. Jende^6,7^, Khalaf Bushara^8^, Leire Manrique^9^, Pauline Lallemant-Dudek^10^, Sandro Romanzetti^11^, Alexander Lange^1^, Maya Shrestha^1^, Anton Ludwig^1^, Alena Rosenow^1^, Tim Elter^1^, Magda M. Santana^12,13^, Eberhard Pracht^1^, Tony Stoecker^1^

1. German Center for Neurodegenerative Diseases (DZNE), Bonn, Germany
2. Center for Neurology, Department of Parkinson, Sleep and Movement Disorders, University Hospital Bonn, Bonn, Germany
3. Department of Neurology, Donders Institute for Brain, Cognition, and Behaviour, Radboud University Medical Center, Nijmegen, The Netherlands
4. Department of Neurology and Center for Translational Neuro- and Behavioral Sciences, University of Duisburg-Essen, Essen, Germany
5. Department of Diagnostic and Interventional Neuroradiology, Hospital Tübingen, Tübingen, Germany
6. Division of Radiology, German Cancer Research Center, Heidelberg, Germany
7. Department of Neuroradiology, Heidelberg University Hospital, Heidelberg, Germany
8. Department of Neurology, University of Minnesota Medical School, Minneapolis, MN, USA
9. University Hospital of Navarra, Pamplona, Spain
10. Sorbonne Université, Paris Brain Institute - ICM, Inserm, CNRS, APHP, Pitié-Salpêtrière University Hospital, Paris, France
11. Department of Neurology, RWTH Aachen University, Germany, RWTH Aachen University, Aachen, Germany
12. Center for Neuroscience and Cell Biology, University of Coimbra (CNC-UC), Coimbra, Portugal
13. Center for Innovative in Biomedicine and Biotechnology (CIBB), University of Coimbra, Coimbra, Portugal

# DELCODE/DANCER study group

Falk Lüsebrink^1^, Stefan Hetzer^2^, Michael Ewers^3,4^, Julian Hellmann-Regen^5,6,7^, Eike Spruth^5,8^, Daniel Janowitz^4^, Ingo Kilimann^9,10^, Marie T. Kronmüller^11^, Annika Spottke^11,12^, Oliver Peters^5,8^, Josef Priller^5,8,13,14,15^, Katharina Buerger^3,4^, Stefan Teipel^9,10^, Frank Jessen^11,16,17^, Emrah Düzel^1,18^, Anna Gamez^11^, Hannah Asperger^11^, Okka Kimmich^11,19^, Gabor C. Petzold^11,20^

1. German Center for Neurodegenerative Diseases (DZNE), Magdeburg, Germany
2. Berlin Center for Advanced Neuroimaging, Charité – Universitätsmedizin Berlin, Berlin, Germany
3. German Center for Neurodegenerative Diseases (DZNE), Munich, Germany
4. Institute for Stroke and Dementia Research (ISD), University Hospital, LMU Munich, Munich, Germany
5. German Center for Neurodegenerative Diseases (DZNE), Berlin, Germany
6. Department of Psychiatry and Neurosciences, Charité – Universitätsmedizin Berlin, Berlin, Germany
7. ECRC Experimental and Clinical Research Center, Charité – Universitätsmedizin Berlin, Berlin, Germany
8. Institute of Psychiatry and Psychotherapy, Charité – Universitätsmedizin Berlin, Berlin, Germany
9. German Center for Neurodegenerative Diseases (DZNE), Rostock, Germany
10. Department of Psychosomatic Medicine, Rostock University Medical Center, Rostock, Germany
11. German Center for Neurodegenerative Diseases (DZNE), Bonn, Germany
12. Center for Neurology, Department of Parkinson's Disease, Sleep and Movement Disorders, University Hospital Bonn, Bonn, Germany
13. University of Edinburgh and UK DRI, Edinburgh, UK
14. Department of Psychiatry and Psychotherapy, School of Medicine and Health, Technical University of Munich, Munich, Germany
15. German Center for Mental Health (DZPG), Munich, Germany
16. Department of Psychiatry, University of Cologne, Cologne, Germany
17. Excellence Cluster on Cellular Stress Responses in Aging-Associated Diseases (CECAD), Cologne, Germany
18. Institute of Cognitive Neurology and Dementia Research (IKND), Otto-von-Guericke University, Magdeburg, Germany
19. Center for Neurology, Department of Parkinson's Disease, Sleep and Movement Disorders, University Hospital Bonn, Bonn, Germany
20. Center for Neurology, Department of Vascular Neurology, University Hospital Bonn, Bonn, Germany.
